# Radiation symptoms resemble laminopathies and the physical underlying cause may sit at the lamin A C-terminus

**DOI:** 10.1186/s10020-025-01081-0

**Published:** 2025-02-20

**Authors:** Alexandra Waldherr, Anna Fogtman

**Affiliations:** 1 Max-Planck Institute for Biology, Max-Planck-Ring 5, 72076 Tübingen, Germany; 2 European Space Agency, Space Medicine Team, EAC European Astronaut Center, EAC Linder Höhe, 51147 Troisdorf, Germany

## Abstract

Ionizing radiation causes three divergent effects in the human body: On one side, tissue death (= deterministic effects) sets on, on the other side, mutations and cancer growth (= stochastic effects) can occur. In recent years, the additional phenomenon of accelerated aging has come to light. In the following, we argue that these seemingly contradictory radiation responses namely: (i) increased cancer growth, (ii) ablation of cancer tissue or (iii) deterministic senescence, share an underlying cause from damage at the lamin A C-terminus. In other words, besides the typically described genomic radiation impact, we propose an additional destabilization pathway via oxidation at the nuclear envelope. We propose five concrete hypotheses that draw a direct mechanistic model from radiation damage and cellular oxidative stress, to micronuclei and clinical symptoms. In conjunction with lamin B compensation, we might be able to explain why deterministic or stochastic responses dominate. If our model holds true, a novel target for radiotherapeutics and radiooncology arises, and a rationale to closer connect laminopathy and radioprotection research.

**Graphical Abstract:**

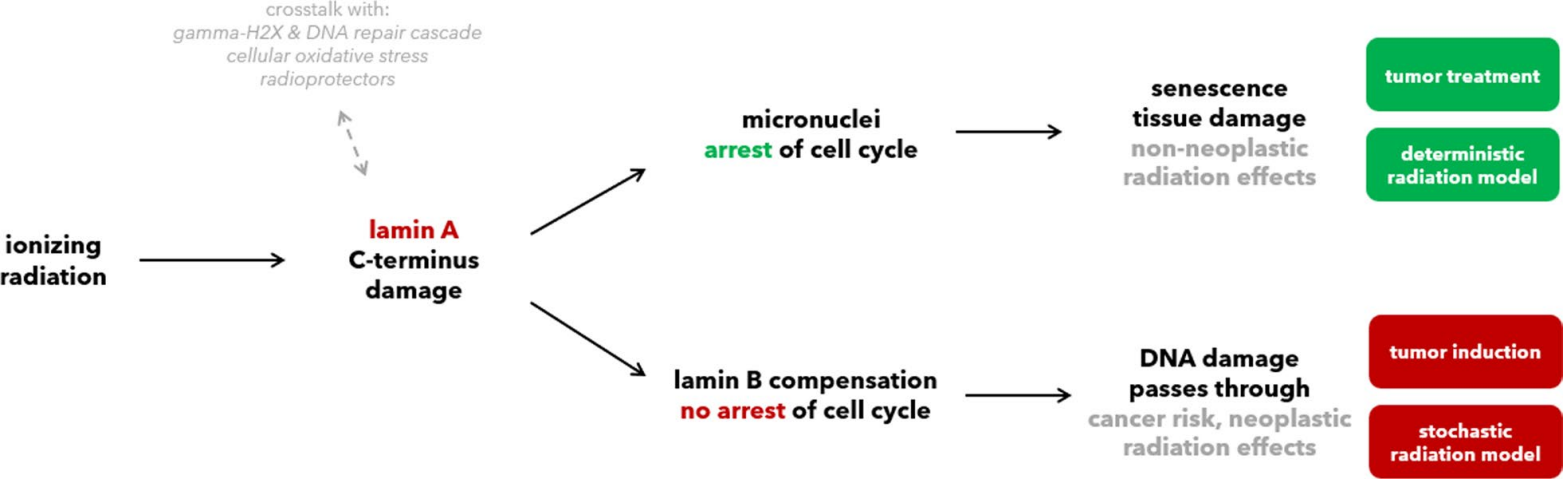

**Supplementary Information:**

The online version contains supplementary material available at 10.1186/s10020-025-01081-0.

## Introduction

Astronauts in space and cancer patients in clinics are exposed to ionizing radiation. High doses lead to acute radiation sickness, while lower doses lead to neoplastic or non-neoplastic effects (Dietze et al. 2013). For example, in exposed astronauts, post-flight Kaplan–Meier curves for cancer mortality vs. cardiovascular disease can be analyzed. These are statistically independent (Reynolds et al. 2019). Cancer, the neoplastic endpoint, versus cardiovascular damage, a typical non-neoplastic endpoint, underlie two different cellular responses, otherwise radiation could not attribute for statistically different endpoint response.

Genetic conditions further underline nuances in response. Mutations in *ATM* (ataxia telangiectasia), *TP53* (Li Fraumeni’s syndrome) or *ERCC6/8* (Cockayne’s syndrome) come with a different profile of heightened radiosensitivity and/or radiosuspectibility (El-Nachef et al. 2021; Foray et al. 2016; Deschavanne and Fertil 1996).^1^^*^ However, direct cellular actors impaired apart from DNA remain poorly illuminated.

With the current model, DNA double strand breaks (DSB) and their repair are in focus of radiation biology, ATM being one key actor. As a cytoplasmic protein, it monomerizes after irradiation, translocates to the nucleus and recognizes DNA damage sites. At these foci, ATM phosphorylates downstream actors like histone γH2AX by which repair sites can be counted using immunofluorescence.

Foray et al. were able to use the speed of ATM shuttling to establish the RIANS model (*radiation-induced ATM nucleo-shuttling*). Individuals can be classified thereafter in Group I (normoresistant, fast shuttling), Group II (moderate radiosensitivity, delayed shuttling) and Group III (hyperradiosensitive, inhibited shuttling) (Berthel et al. 2019; Foray 2020).

**Fig. 1:**
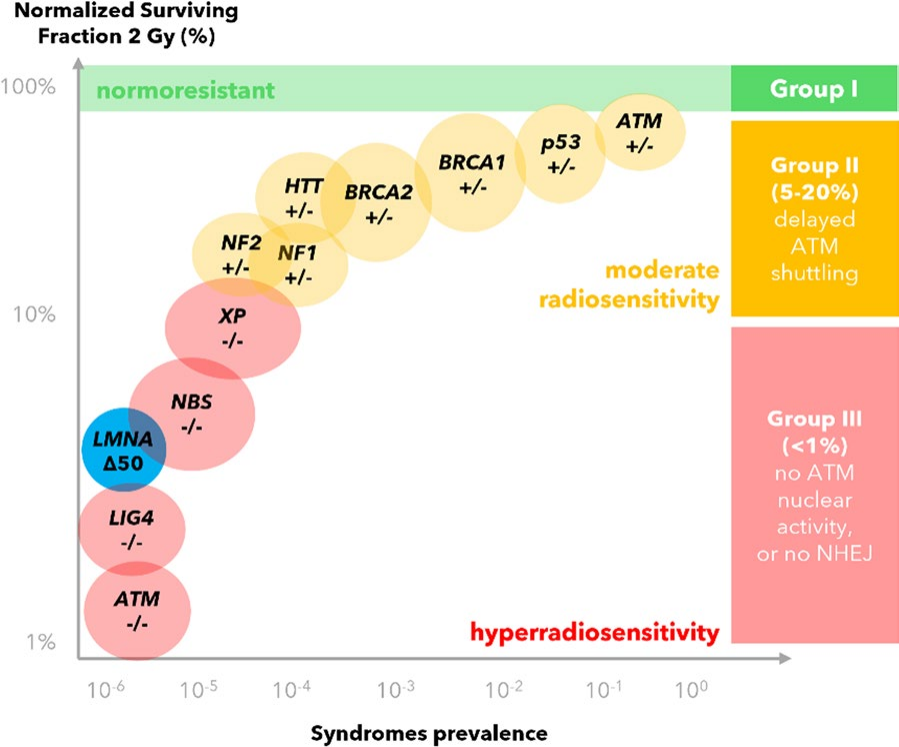
Classes of radiosensitivity and impairing genotype by the RIANS model. Shown are fibroblast surviving fractions. Axes are labeled logarithmically. Progeria patient cell data is marked in blue. Data after Foray et al. and Deschavanne et al. (Deschavanne and Fertil 1996; Berthel et al. 2019)

Specific genotypes have been identified per group, summarized in Fig. 1.

For the course of this work, three terms will be used and justify an initial definition: Radiosensitivity (= tissue reaction), radiosuspectibility (= neoplastic risk) and radiodegeneration (= aging effect).

*Radiodegeneration* and *radiosensitivity* are not always differentiated. *Radiosensitivity* is the common term used to describe the likelihood for *non-cancerous* symptoms to appear in an individual after irradiation, often attributable to cell death. *Radiodegeneration* is a much less used term, often to separate responses that mimic aging not attributable to cell death alone, *e.g*., cataracts or cardiovascular risk. We hope to increase awareness for the term with this work. Both terms together count for the *non-neoplastic* risks, which sets them apart from *radiosuspectibility*, used on *cancer* predisposition (El-Nachef et al. 2021; Foray et al. 2016).

At ESA, we are currently invested in elucidating the suite of non-cancerous radiation effects to protect astronauts by targeted and early interventions better.

Non-neoplastic effects appear seemingly unrelated and present across almost all organ classes, *e.g.* cardiovascular risk, cataract, neurological shifts, inflammation, signs of lipodystrophy. As microgravity is a confounder, we extracted only those biological shifts that are matched in exposed gravity-bound 1G cohorts (NASA 2016; NASA's Twin Study Results Published 2019; Space Radiation Risks 2024; Visentin et al. 2019). While between these symptoms no logical clinical connection seemed present, an overlap to the phenotype of *progeria patients* became to us strikingly apparent.

**Fig. 2:**
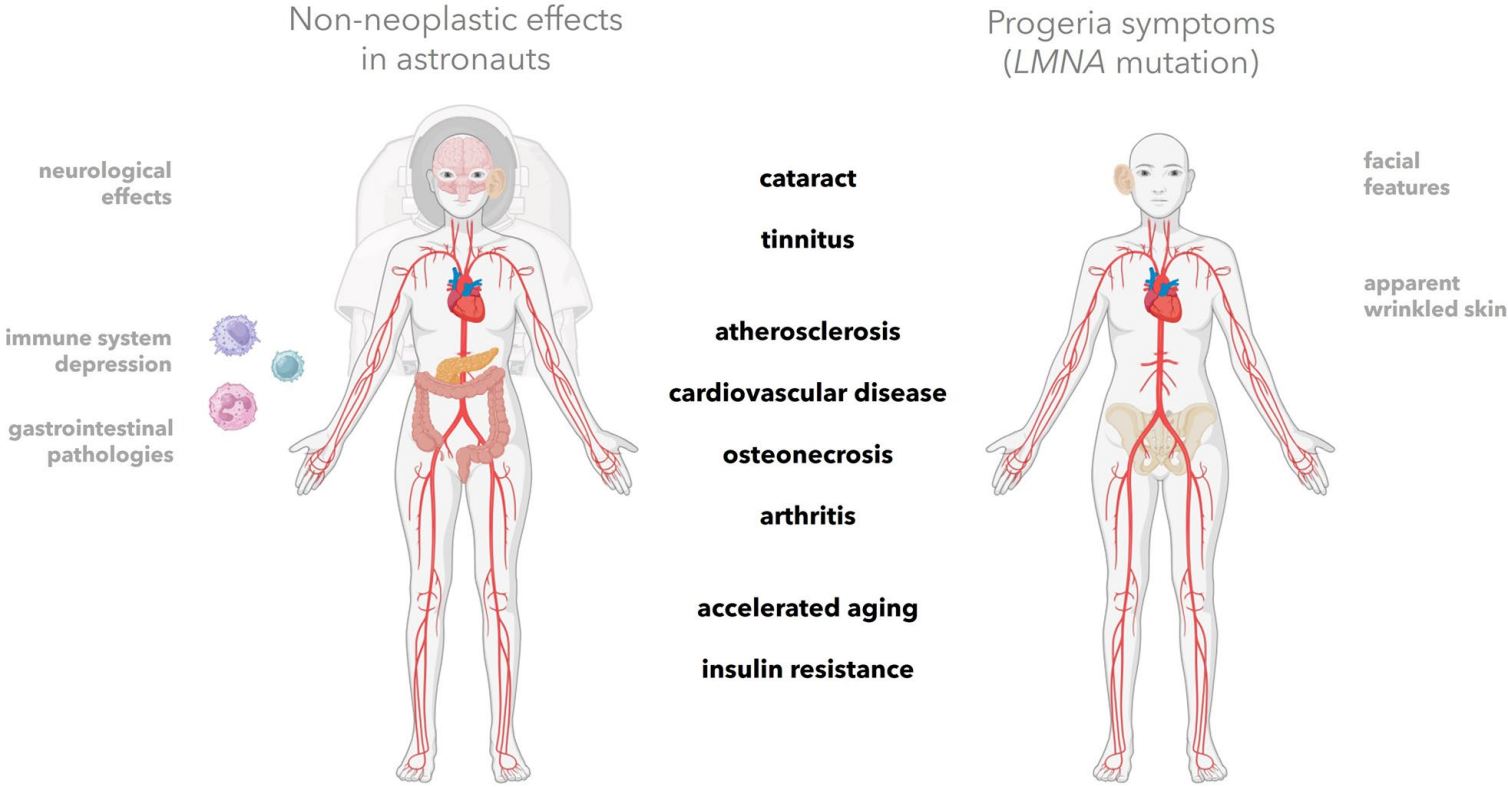
Parallels of the non-neoplastic effects observed in exposed astronauts and 1G-cohorts (left) compared to Hutchinson-Gilford progeria (right). A large set of symptom overlap (black, middle). A few pathologies have been only reported in either astronauts or progeria patients, (grey, only left or only right). The large symptom overlap motivated us to model the possibility of a “space” or “radiation laminopathy”. Created in BioRender. Arreba, P. (2025) https://BioRender.com/v77b421

In the other direction, progeria patients are also highly radiosensitive (RIANS Group III, Fig. 1) (Foray 2020; Redwood et al. 2011). Inspired by this observations in bilateral direction, progeria $\stackrel{\phantom{a}}{\iff }$ radiation symptoms, we embarked on investigating whether radiation may trigger pathophysiology through pathways directly impairing lamin A (Fig. 2).

### Hutchinson-Gilford progeria and astronaut similarities

Hutchinson-Gilford progeria patients show a typical phenotype of rapid accelerated aging. The underlying mutation, a silent mutation in *LMNA* (*c.C1824T* G608G), leads to a splicing defect, resulting in the translation of the truncated *progerin*. This mutant lamin A variant lacks Δ50 amino acids at its C-terminus, including the cleavage site for the protease ZMPSTE24 (Eriksson et al. 2003). Lamin A typically matures per C-terminal farnesylation and subsequent C-terminal cleavage by ZMPSTE24. Instead, a pool of constantly farnesylated Δ50 lamin A accumulates at the nuclear membrane (Goldman et al. 2024; Cleveland Clinic 2022).

Lamin C, also encoded by *LMNA*, shares the first 566 amino acid but is spliced to a shorter variant and does not undergo as extensive posttranslational processing.

Mature lamin A interacts with a set of proteins at the inner nuclear membrane and inside the nucleoplasm, among other tasks regulating heterochromatinization. Progerin remains constantly farnesylated at the inner nuclear membrane. This causes a dramatic deformation of the nucleus, with blebs and lobular features. Associated clinical symptoms are cardiovascular effects, cataracts, atherosclerosis, osteonecrosis, and diabetes (Eriksson et al. 2003; Cleveland Clinic 2022; Kato and Maezawa 2022; Holder and Schwitzgebel 2020). Children with progeria die early and mostly from cardiovascular problems such as heart attack or stroke.

In adult astronauts, very similar clinical symptoms are observed after spaceflight and most are not yet understood. These include cardiovascular dysfunction, immune system dysfunction, cataract, induced insulin resistance, osteonecrosis, arthritis, neurological damage and accelerated aging (Dietze et al. 2013; Space Radiation Risks 2024; European Space Agency, Space Medicine Team 2022). Latter was prominently demonstrated in a twin study between astronaut Scott Kelly and Mark Kelly (NASA's Twin Study Results Published 2019). While attributes are cofounded by microgravity, the symptoms which overlap with progeria agree with those symptoms found in the exposed workers or radiotherapy cohorts on Earth, *e.g.*, mandibular osteonecrosis in dentistry, cataract, cardiovascular dysfunction, inflammation, sensitivity of the brain (European Space Agency, Space Medicine Team 2022; Huff et al. 2022).

On the cellular level, hallmark symptoms of irradiation are increased oxidative stress, micronuclei formation and DNA damage and repair dysregulation (European Space Agency, Space Medicine Team 2022; Little 2003; Azzam et al. 2014). The cause are two ways in which ionizing radiation damages macromolecules: (i) through direct impact (*e.g.* DNA strand breaks) or (ii) through secondary effects after radiolysis of water, where O–H bonds get broken and reactive oxygen species (ROS) created that oxidize macromolecules (Reisz et al. 2014).

### Five steps towards a mechanistic model

From the observed clinical similarities between progeria and irradiation, we started to examine the nuclear lamina in context of radiation. Especially the C-terminus of lamin A, affected in progeria, marked a central point of interest. We could pinpoint down three conserved cysteines (C522, C588, C591) that get readily oxidized and from there established a model of *space* or *radiation laminopathy* in five steps. The model puts the nuclear lamina more into focus than previous models, and can complement the RIANS model.

Before introducing the five hypothesis, the nuclear lamina's function is briefly summarized:

The nuclear lamina consists of a fibrillar network at the inner side of the nuclear envelope, only present in mammalian cells. Lamins A/C (*LMNA*) and B-type lamins B1 (*LMNB1*) and B2 (*LMNB2*) are the primary structural elements, and themselves form coiled-coil tetramers, similar to cytosolic intermediate filaments. Lamin B1 and B2 reside only perinuclearly, and Lamin B1 can regulate transcription of oxidative stress responders via Oct-1 (Malhas et al. 2009). Lamin A, on the other hand, is fixed at the envelope to form LINC complexes with SUN proteins and nesprins to the cytoskeleton outside, but it can also transitions to an active states inside the nucleoplasm. Numerous mutations in *LMNA* have been found to cause human diseases, one of which is progeria, collectively termed *laminopathies*. These pathotypes uncovered various functions for lamin A, including stiffening of the nuclear architecture, and regulating processes of DNA synthesis and repair (Goldman et al. 2024; Shimi and Goldman 2014; Paul and Fulka 2022).

Evidence suggests an interrelationship between lamin A and ATM:

Mahen et al. showed that lamin A stabilizes DNA damage repair foci through engaging ATM-phosphorylated γH2AX (Mahen et al. 2013)and Gibbs-Seymour et al. demonstrated that 53BP1, an ATM upstream activator and substrate, binds to lamin A via its Tudor domain while *LMNA* -/- mutants exhibit a 53BP1 null phenotype (Mochan et al. 2004; Gibbs-Seymour et al. 2015). Recently, Kim et al. defined the subcellular location of early DNA response proteins for the three kinases (ATM, ATR, DNA-PK) and the MRN complex (MRE11-RAD50-NBS1) and a lack of lamin A/C impaired (i) IR-triggered interaction of ATM an MRN, (ii) association of ATM with the nuclear matrix, and (iii) orchestrated DNA repair (Kim et al. 2024). In the other direction, Shah et al. showed that ATM deletion led to a reduced lamin A expression (Shah et al. 2022), as is the case in radiosensitive ataxia telangiectasia patients.

Together, these reports supported our view that lamin A may be part of the radiation response.

Lastly**,** ATM activation itself through oxidative (ionizing) stress is achieved via cysteines. Howes et al. structurally resolved the ROS-activated form of ATM and it is a disulfide-bound dimer with p53 as substrate (Guo et al. 2010; Howes et al. 2023).

The strong evidence by Howes et al. puts the completeness of the current RIANS model into question, which relies on monomerization of cytosolic ATM. Autophosphorylation is proposed, which for ATM however is mainly observed in the nucleus under MRN complex dependency (Kozlov et al. 2015). In the following, we introduce lamin A to the picture and two possible stages of oxidative stress, the first promoting disulfide bonds and later causing futile sulfonic species.

To be sure, we think of our model as initial starting point to include the nuclear envelope in radiation biology. There are many unknowns, we propose following five hypotheses to test:

1. The first hypothesis is that secondary radiation damage oxidizes and damages lamin A at its C-terminal C522, C588 and C591, three highly conserved cysteine residues. These residues lie close to an Ig-fold domain which interacts, *e.g.*, with heterochromatin and $\gamma$γH2AX anchor sites (Kim et al. 2024; Gonzalo 2014). The oxidation may initially proceed to functional disulfide bridges, but under too strong irradiation to higher species cause lost functionality. We suggest that shifts from disulfide activation to sulfonic acid deactivation could equate to threshold doses. Oxidized lamin A possibly accumulates perinuclearly, and is known to restructure the nuclear envelope, leading to laminopathy-like dysmorphic nuclei (Pekovic et al. 2011).
2. As second hypothesis, we review that islands of high lamin A in nuclear membranes oppose nuclear pore complex formation. We conjecture that accumulated, damaged lamin A could impact the speed of ATM in this way, advancing the RIANS model.
3. As third hypothesis, we review how micronuclei can have high levels of lamin A, increasing likelier rupture and activating the cGAS-STING pathway. In opposition stands activation of the apoptotic cascade through oxidative stress. We project from this why cell types with higher lamin A seem more buffered to acute radiosensitivity but may suffer consequences of cGAS-STING in the form of radiodegeneration.
4. Our fourth hypothesis is that cell types with higher lamin B1/2 can bypass both effects. In such cell types, lamin B1 can aid oxidative stress response, but at the cost of higher risk for radiosuspectibility for cancer. High lamin B1 could allow cells to stay in the cell cycle but with more malleable nuclear envelopes and instable genomes. As first slight indicator, we show *Human Protein Atlas* expression levels in normal and cancer cell lines (Etourneaud et al. 2021; Lv et al. 2024a; Evangelisti et al. 2022).
5. Lastly, we address the phosphorylation of lamins. We present SQ/TQ motives, phosphorylation sites for ATM and ATR, which connect our model back to the known damage response kinases. Secondly, we discuss phosphorylation of lamins in the context of mitosis. We propose that rapidly dividing cells (*i.e.* compare radiation therapy) may be most susceptible to ionizing radiation not only because of the accumulating genomic errors, but due to the requirement of disassembly and correct reassembly of the nuclear envelope in *G2/M* stages. Damage of lamins would inhibit this process, killing cells at high fractions and effectiveness. This could have vast implications for radiooncology.

#### Hypothesis 1: The molecular “radiation injury” of lamin A

We started from possible radiation damage modes on macromolecules and reviewed known classes of radioprotectors along their chemical properties. From this, we identified vulnerable lamin A residues. The most expansive database currently available is RadioProtectors (Aliper et al. 2020). It lists 186 chemical structures with proposed mechanism of action and source publications.

To date, radioprotector classes are still discovered empirically rather than designed rationally. The first radioprotector found was cysteine in 1949, and since then the class of sulfhydryl compounds (-SH) has comprised the largest class of radioprotectors. It includes the most potent radioprotectant, amifostine (Aliper et al. 2020).

Therefore, we filtered for cysteine residues (-SH). They present equally as sulfhydryl compounds, and as obvious endogenous targets for reactive oxygen species in irradiated cells.

**Fig. 3:**
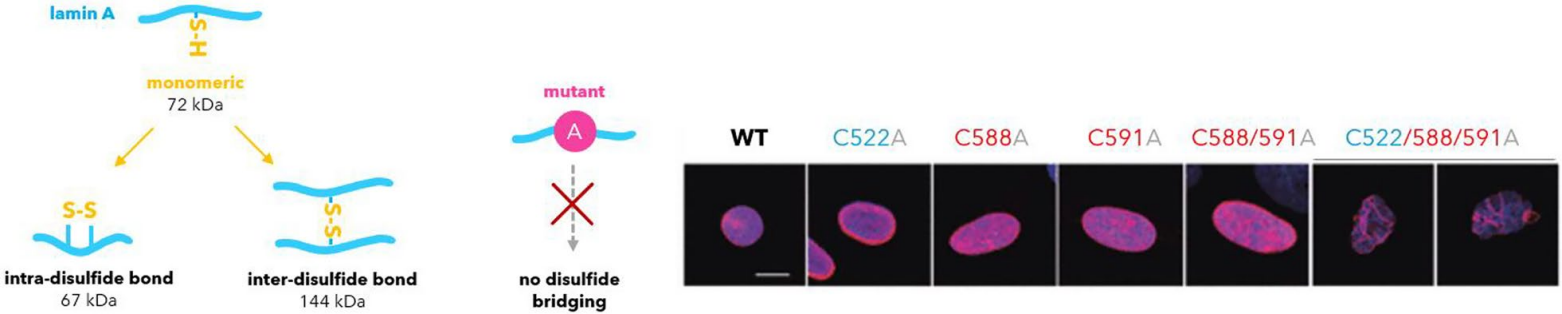
Lamin A cysteine residues enable intramolecular and intermolecular disulfide bridging (left). Absence of cysteine residues in cysteine-to-alanine mutants mimics progeria. Mutants respond by peripheral relocalization (C522A) to complete membrane restructuring (C522/588/591). Lamin A in pink. Reproduced after Prekovic et al. (2011)

Three highly conserved cysteine residues were found in the lamin A C-terminus: Inside a immunoglobulin-like fold, C522, and conserved among all vertebrates, C588 and C591. Current evidence reports their direct involvement in cellular responses to reactive oxygen species. Prekovic et al. showed that these cysteines establish disulfide bonds, which are directly impaired under oxidative damage. Damaged cellular features were dysmorphic nuclei and cellular senescence (Pekovic et al. 2011). They validated their findings in cysteine-to-alanine mutants, which responded by localizing the damaged variant to the nuclear membrane, see Fig. 3. This correlates to the pathophysiology of progeria.

**Fig. 4:**
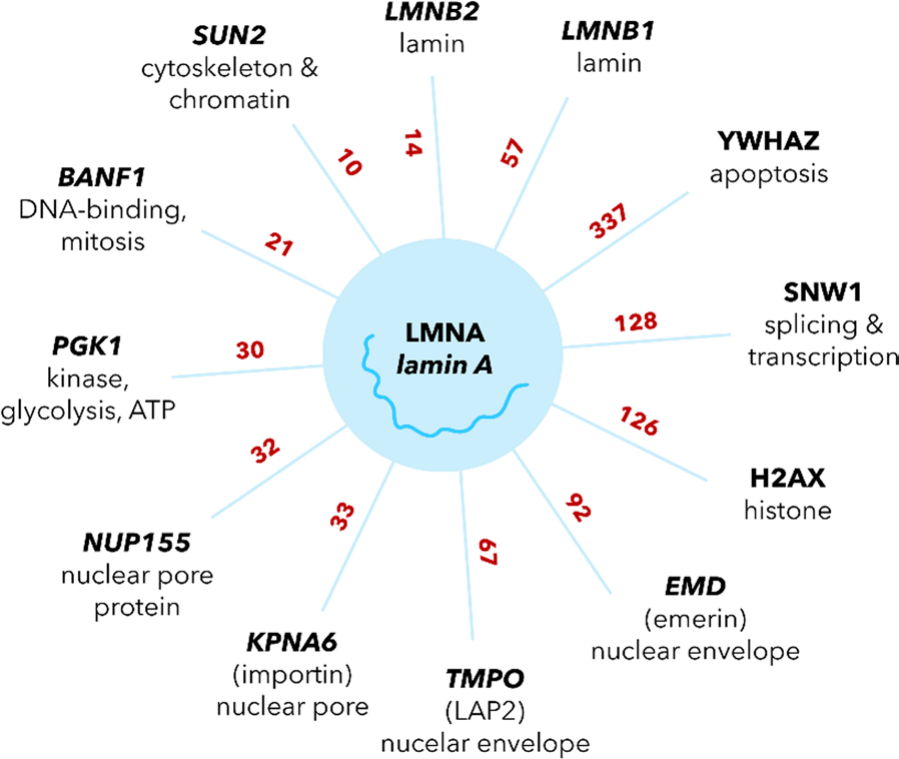
Other lamin A interactions (proteins named after their gene, data adapted after BioGrid (BioGRID. 2025; Atlas et al. 2024). Number of interactions experiments recorded in the databank in dark red. Interaction partners with 10 validating entries or more are shown. Each interaction partner with brief notion of function or location

The question of whether oxidation causes a gain-of-activity or a loss-of-function may be dependent on the oxidative level:

On one side, under physiological conditions, the cysteines C522, C588 and C591 are present in oxidized intra- and oxidized disulfide (S–S) states with high functional importance for binding interactions. Close to the cysteines, lies the Ig-fold (C522 is part of it) which is a central hub for binding interactions. Stierlé et al. showed that the Ig-fold binds DNA after dimerization via disulfide bridges (Stierlé et al. 2003). Many protein partners have also been mapped to the Ig-domain. For example, Lamin A can directly bind proliferating cell nuclear antigen (PCNA), and we refer to a small overview in Fig. 4 (Wallace et al. 2023; Shumaker et al. 2008; Thanisch et al. 2017, BioGRID. 2025).

**Fig. 5:**
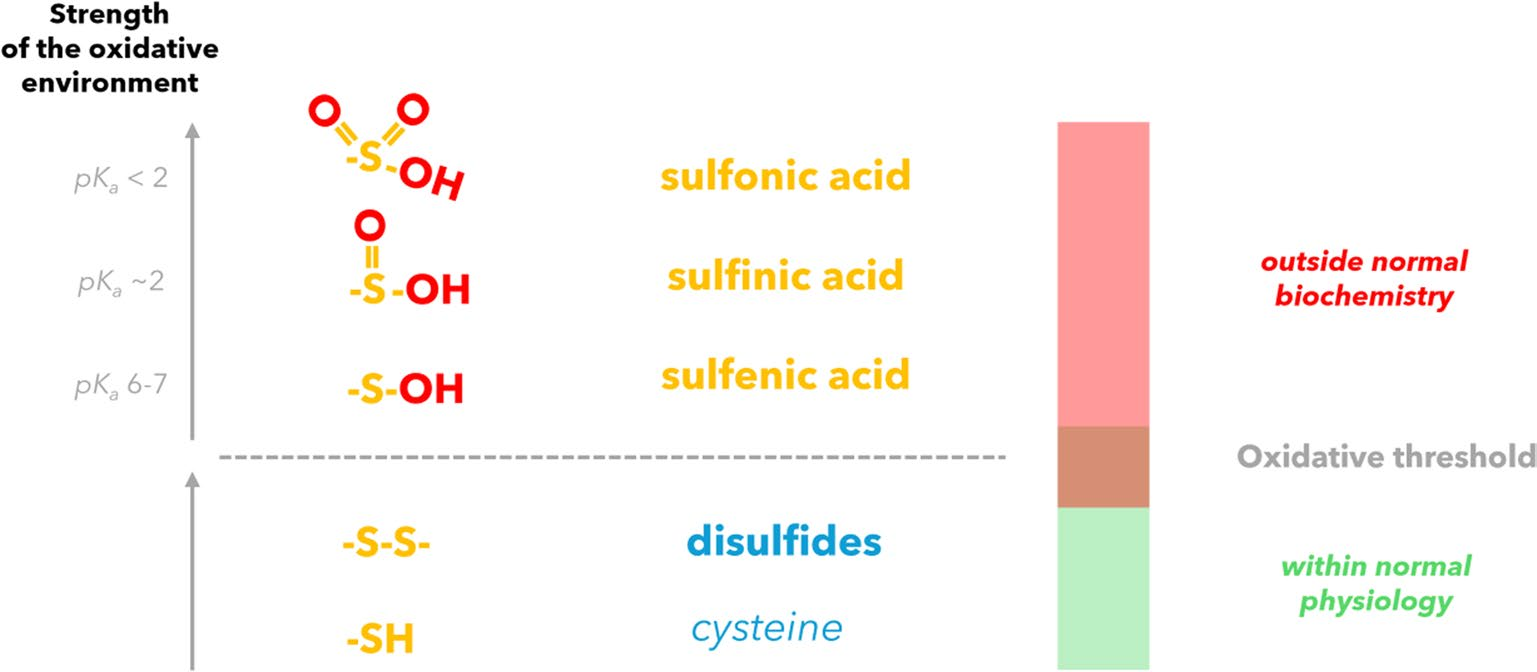
Threshold doses could correlate with the strength of the oxidative environment they induce in cells. Depending on specific proteins and the local environment per cysteine, lower oxidative stress encourages disulfide bonds—a normal range [as found for the activation of ATM (Mochan et al. 2004)]—while higher levels cause oxidation towards unphysiological states. pK_a_ thresholds after Ruiz et al. (2022)

Opposed to disulfide bridging, extreme levels of ROS would oxidize the cysteines too far. This would render them futile to establish a disulfide bridging, compare Fig. 5.

**We suggest there is a two-staged oxidation process:**

**Fig. 6:**
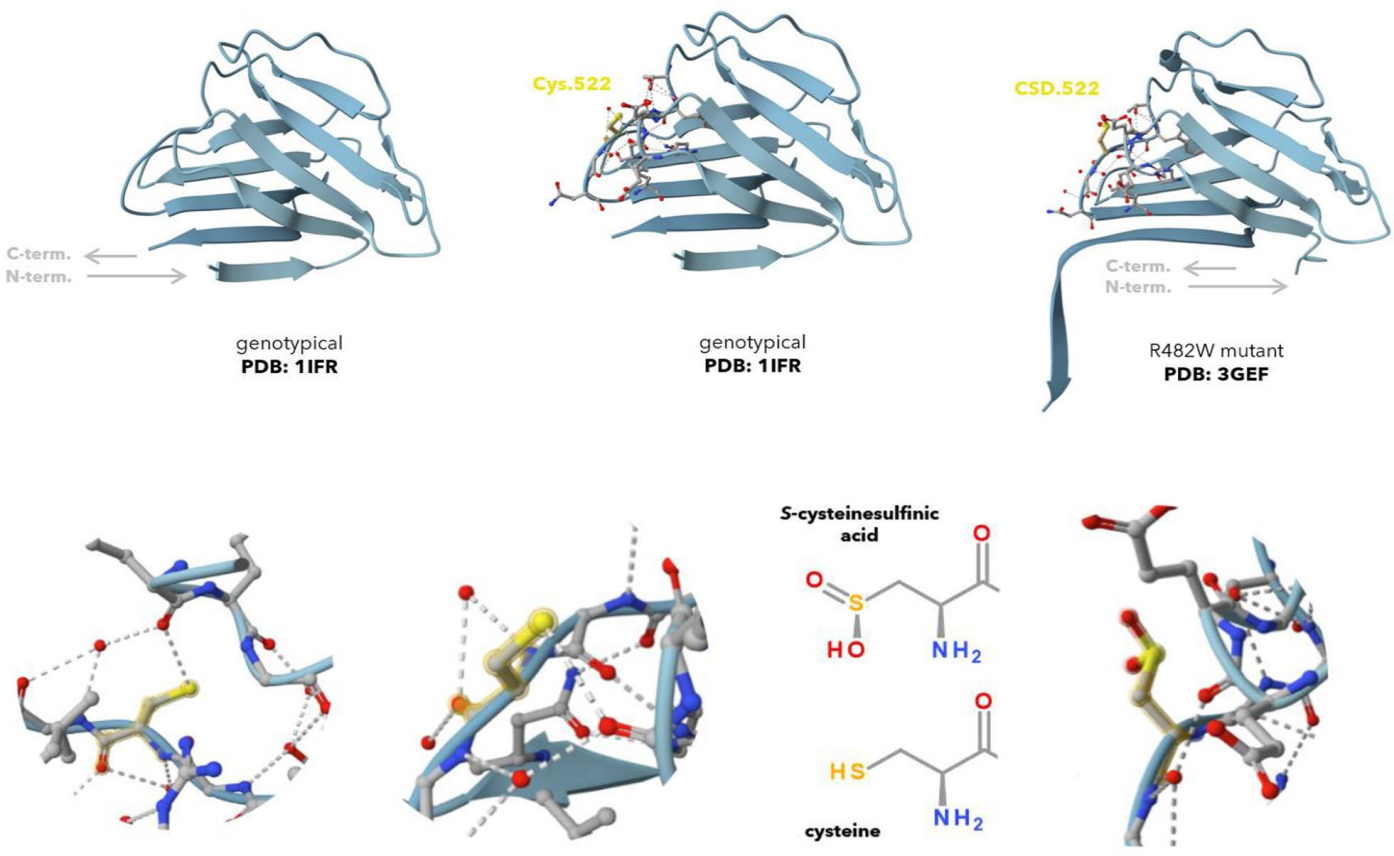
C522 and the oxidized form CSD.522 (*S*-cysteinesulfinic acid) in resolved substructures, PDB data (Bioinformatics and for Structural. RCSB PDB - Protein Data Bank. 2025). In the genotypical lamin A Ig-fold (PDB: 1IFR (2002) top left), the unoxidized C522 lies in proximity to trimmed C-terminal continuation and integrated in the Ig-fold with interaction possibilities to the backbone around E447 (bottom left, bottom middle). In the R482W mutant form (PDB: 3GEF, (2009) top right) present in lipodystrophy laminopathy patients, the residue did oxidized during crystal formation as reported by experimenters. Partnering inside the Ig-fold is diminished, the CSD.522 extended more towards the surface (bottom right, visualized in top middle, top right)

(i) Lower levels of ROS (equivalent to the activation of ATM into disulfide-bound form) could positively enforce disulfide bonds and lamin A activity.
(ii) Higher levels of ROS, above a certain threshold, would exceed oxidation and rather cause sulfonic acid species. The cysteines would be rendered into a damaged and futile form for binding, accumulating like C > A mutants or progerin at the envelope (Fig. 6).

This might explain radiation dose threshold phenomena, also for other proteins like ATM, with active disulfide-bounded states. Such extreme oxidation outside normal biochemistry were already recorded for C522 in *Homo sapiens* Ig-fold crystal structures.

### Structural modeling of lamin A

No complete lamin A structures are available. To our knowledge, the N-terminal helical coils and the Ig-fold are resolved (PDB: 6JLB, 1IFR, 3GEF). For the Ig-fold, a genotypical (PDB: 1IFR (2002)) and a R482W laminopathy variant (PDB: 3GEF (2009)) can be compared.

In the genotypical Ig-fold, C522 sits unoxidized inside the Ig-fold and interacts with the backbone around E447. Unintended by the experimenters, the same cysteine in the mutant model is recorded only in oxidized form, CSD.522 (S-cysteinesulfinic acid) (Magracheva et al. 2009). Compared to the genotypical structure, CSD.522 sits more off-set and exposed. While one cannot conclude that mutant variants favor oxidation, or oxidized residues are preferably exposed, the two structures provide comparators examples showing that C522 is readily oxidized.

To investigate such possible damage at all three cysteines, we prepared AlphaFold *in-silico* mutant models. We set out to predict what change each damaged cysteine could inflict on the lamin A structure, and if orthologs with mutations at these sites have different radioresistance.

**Fig. 7:**
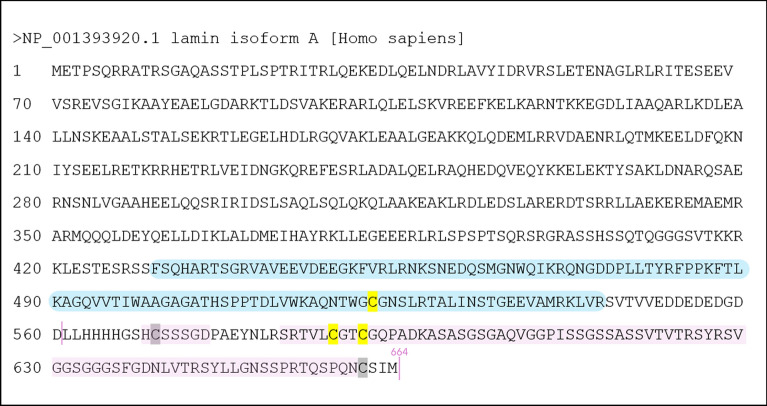
Lamin A protein sequence (Homo sapiens, NP_001393920.1). The Ig-fold is highlighted in blue [after Krimm et al. (2002)]. The conserved cysteine residues are marked in yellow. Two other cysteines present are marked in grey, the -CSIM marks a -CAAX motif for posttranslational processing and is cleaved by ZMPSTE24. In pink, discrepancies in alignment to lamin B1 (Homo sapiens, NP_005564.1, see Supplementary Information) are highlighted, specifically the longer 78-amino acid stretch containing C588 and C591

Lamin A orthologs from eleven species where a radiation sensitivity threshold in LD_50/30_ dose was reported, were aligned. For *Homo sapiens*, the sequence is depicted in Fig. 7. The Ig-fold shown in blue, the cysteines highlighted in yellow.

The Mongolian gerbil is the most radioresistant vertebrate (*Homo sapiens* LD_50/30_ = 400 cGy, *Meriones unguiculatus* LD_50/30_ = 1000 cGy) and was the only to present a mutation, with the eq.C522 changed to a T. This supported our hypothesis, suggesting that while disulfide bonding ability can be lost, the C > T mutation might render *Meriones unguiculatus* more radioresistant.

We further included lamin B1 and lamin B2 in our analysis. B-type lamins hold similar Ig-like fold but have a shorter total length with fewer cysteines. Lamin B1 holds only one C-terminal cysteine (C444, equivalent to C522), while both C588 and C591 are absent. All three phylogenetic trees of lamin A, lamin B1 and lamin B2 mimicked the order of the LD_50/30_ dose sensitivity for the eleven species (see Supplementary Information).

**Fig. 8:**
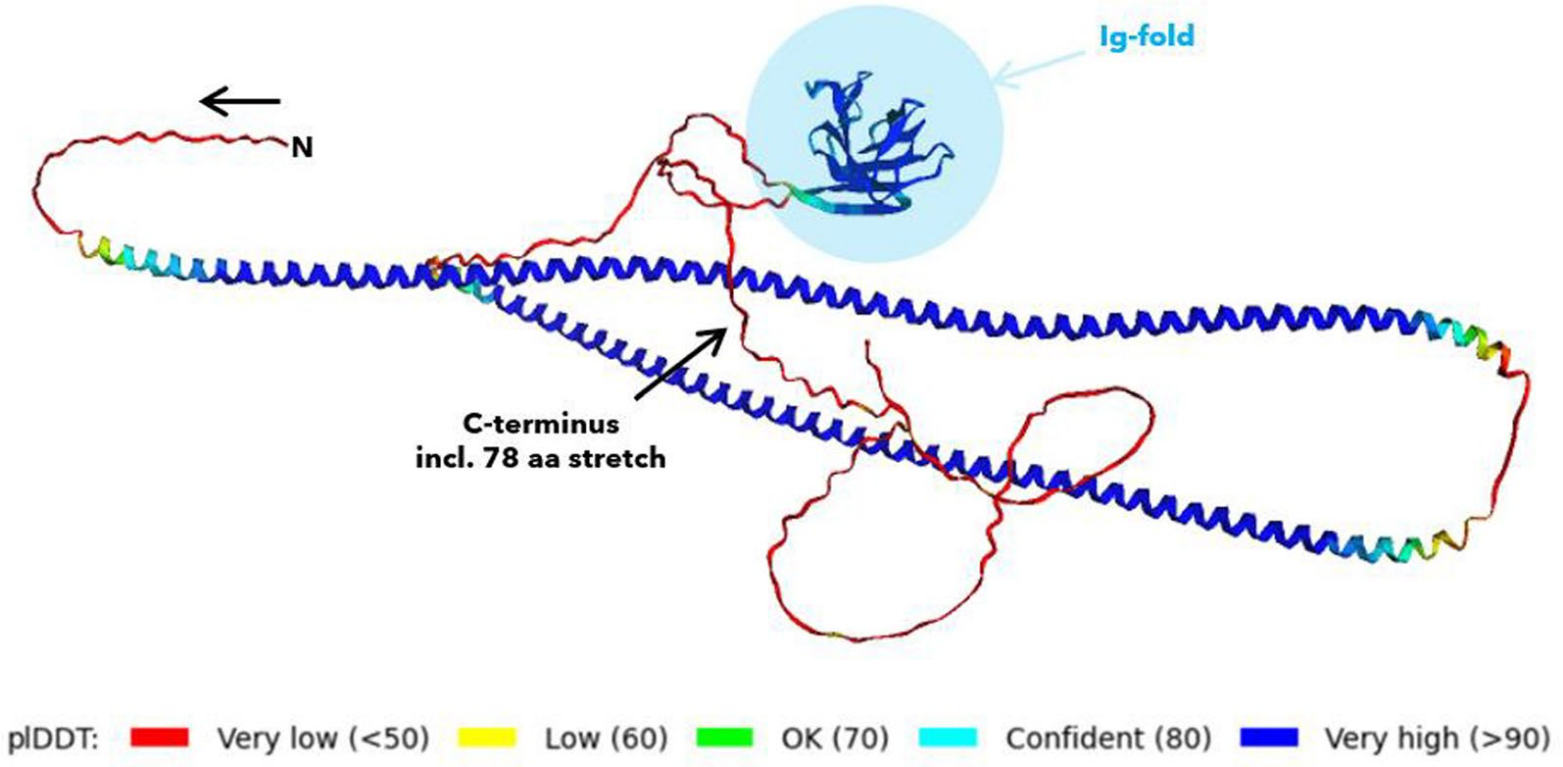
Lamin A protein monomer prediction (Homo sapiens, from NP_001393920.1). By AlphaFold2, including the per-residue confidence estimate (plDDT). The C-terminal 78-amino acid stretch of interest is predicted at low confidence (red, black error). The prediction should not be interpreted after DeepMind and EMBL guidelines, likely exhibiting unresolved or high structural disorder (DeepMind 2021). The Ig-fold is highlighted blue, red ribbon preceeding Ig = NLS (= nuclear localization sequence)

We next modeled the total lamin A sequence to resolve the 78 amino acids that follows the Ig-fold but have never been structurally prepared (Stierlé et al. 2003). This stretch includes C588 and C591, which are conserved among all vertebrates. This structural knowledge missing is curious for such highly conserved residues in a clinically important protein. Our AlphaFold monomer models quickly suggested that one reason for these efforts missing is the high disorder of the stretch, compare Fig. 8 (DeepMind 2021).

We therefore moved from monomer predictions to tetrameric analysis. A tetrameric packing can be expected, as for any intermediate filaments. Lamin A can be submitted for multimer packing to the AlphaFold3 webserver (DeepMind and Server 2024). We submitted the native *Homo sapiens* sequence of lamin A, a triple C > A mutant and three single C > A mutations, as well as the highly radioresistant *Meriones unguiculatus* lamin A sequences for comparison.

In the tetrameric arrangement, the situation around the three cysteines could be more confidently predicted. Specifically, two observations infer structural and functional cruciality of the cysteines: First, the C-terminus becomes clearly positioned outwards, especially in native configuration. This opens it up for binding interactions. Secondly, the C-terminus packs in close proximity to and around the Ig-fold, increasing the likelihood of being a determining factor in binding partner interactions.

**Fig. 9:**
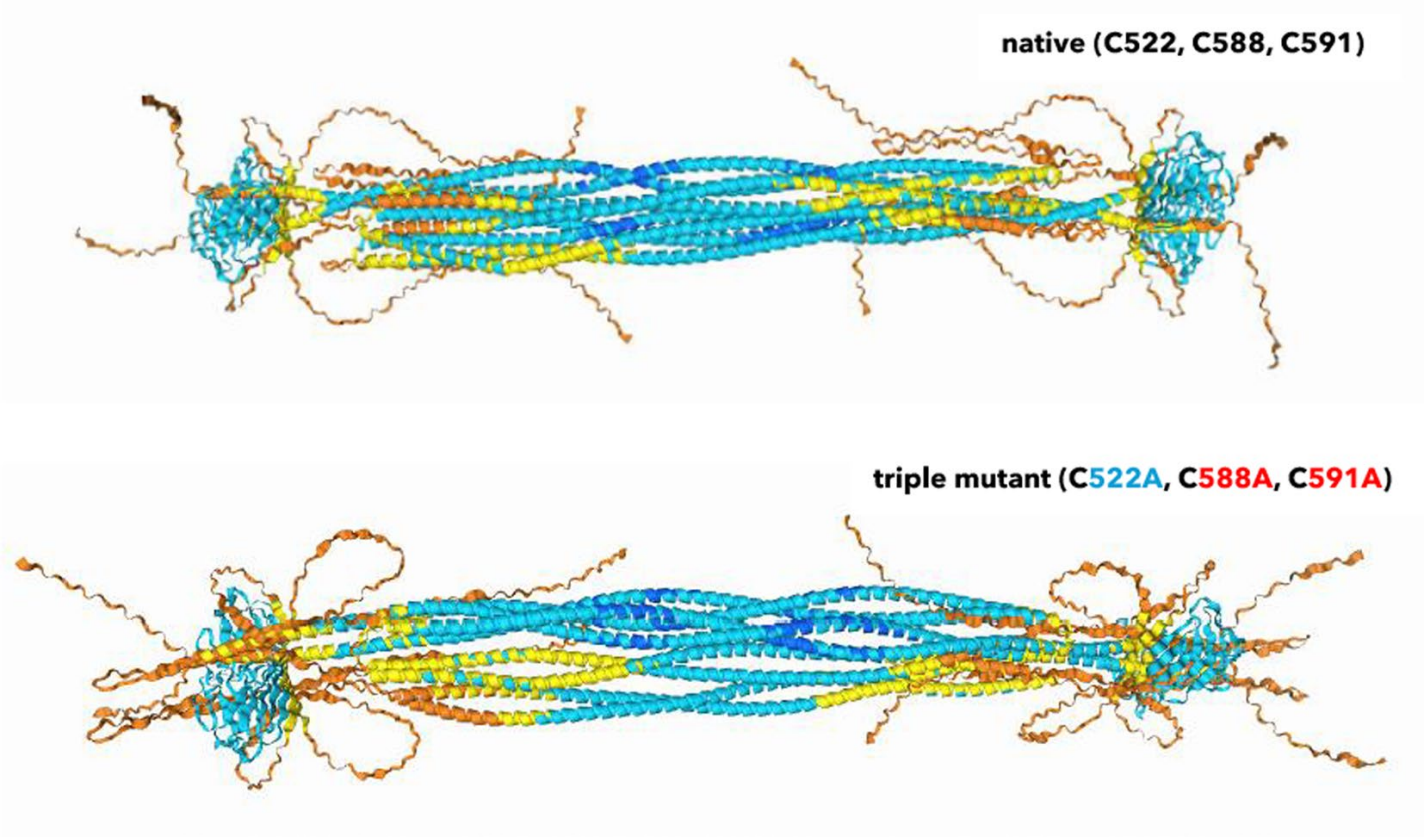
Comparison of the *Homo sapiens* lamin A (native form, top) and a triple C > A mutant (bottom) without C522, C588 and C591 disulfide bridiging possibility. AlphaFold3 (DeepMind, AlphaFold3 Server 2024) predictions show a reshaping of the C-terminal loop from an open configuration into a butterfly-like arrangement. The three cysteines may have crucial influence on an open structure. In C > A mutation without disulfide bonds, other residues dominate and form more confined arrangement without the same possibility for interaction, and Ig-fold interactions

When removing (“oxidizing”) the cysteine via *in-silico* triple mutation C > A, the open configuration becomes shaped into a butterfly-shaped loop, compare Fig. 9.

**Fig. 10:**
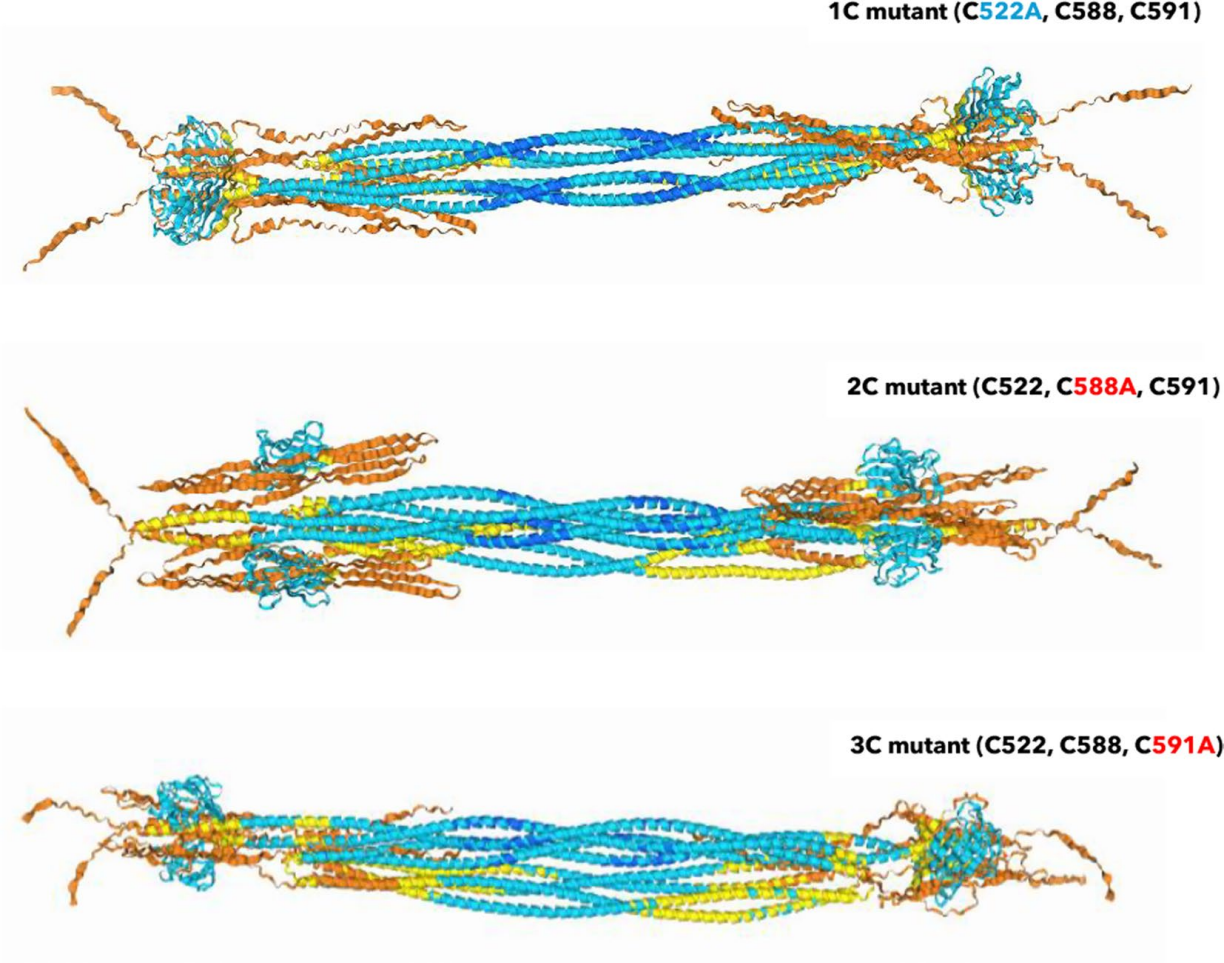
Comparison of the three single *Homo sapiens* C > A *in-silico* mutants. Each mutation impacted the AlphaFold3 prediction quite drastically. *In-vitro*, each single mutant (Fig. 12) showed equally a different effect

Further, each cysteine mutant exhibits different *in-silico* effects. When removing one of the three cysteines, a dominant disulfide bond between the other two can be expected. In other words, if C522 inside the Ig-fold is removed, we expect the C588 and C591 in the flexible C-terminus to exclusively interact. If the C522 position stays intact, but one of the C588 or C591 residues is mutated, we expect the C-terminus to bind farther (C588A, C591 remains) or shorter (C588 remains, C591A) to the Ig-fold. The three *in-silico* mutant structures are shown in Fig. 10.

This expected behavior was observed in the *in-silico* AlphaFold3 tetrameric predictions.

In the C552A mutant, through the remaining C588 to C591 interaction without contact option to the Ig-fold, the C-terminus closed into exclusively separated folds, into a thin band.

In the C588A mutant, most intriguingly, the far distance from C522 to C591 bridged the remaining amino acids in such a stretched way that the Ig-fold was flipped to the coils.

In the C591A mutant, the binding between the remaining C588 and C522 was less drastic. The Ig-fold remained terminal, the C588 to C522 interaction, however, bound the C-terminus tightly to the Ig-fold, almost inside the coiled-coil core domain.

Regarding single mutations of the conserved cysteines in the animal kingdom, the radioresistant *Meriones unguiculatus* evolved the equivalent of a C552T mutation. We submitted the *Meriones unguiculatus* lamin A sequence for tetrameric AlphaFold3 prediction.

**Fig. 11:**
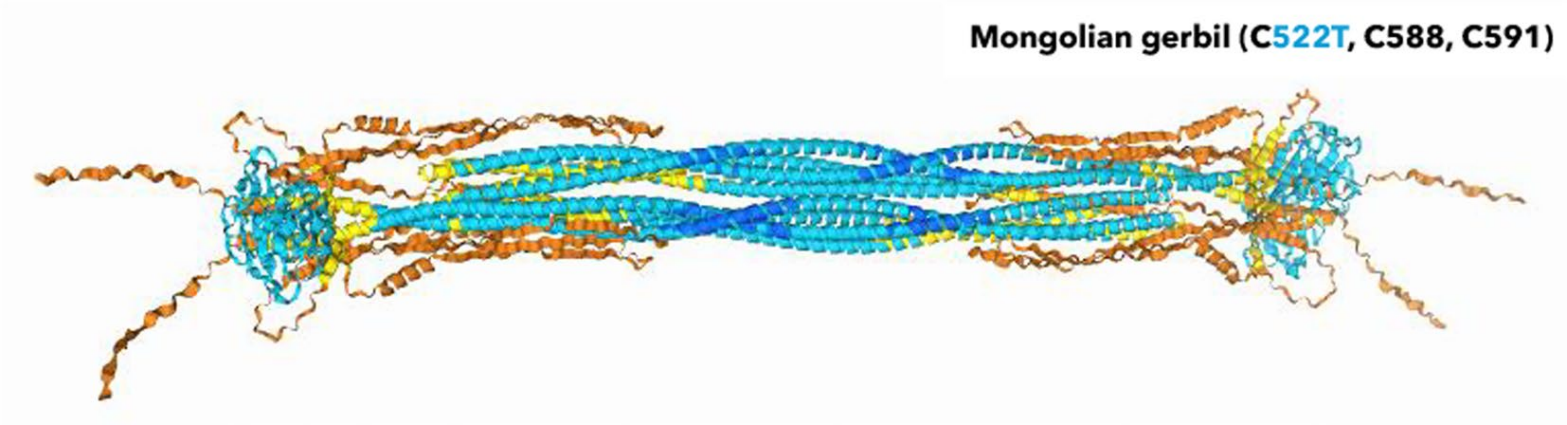
*Meriones unguiculatus* lamin A, equivalent of a C522T mutation. Other mutations evolved in the gene as well when aligned against *Homo sapiens* (sequences see Supplementary Information). These may open up the configuration around the Ig-fold

The prediction assimilated the C552A mutant above. An observed difference of the *in-silico* models was a more open configuration around the *Meriones unguiculatus* Ig-fold, compare Fig. 11 to the human mutants. An immobilization of the C552T mutant at the nuclear membrane, like for the *Homo sapiens* C552A mutant , see/compare Fig. 3 before (Pekovic et al. 2011), may or may not be the case for the Mongolian gerbil. There is no reported data available. Other regions in the protein, and maybe alternative pathways, likely have evolved to counterbalance the C552T mutation while benefitting from a nuclear lamina that is less prone to oxidative stress.

With this, we open *Hypothesis 1* for testing. Interesting for radioprotection would be the investigation of different mutant versions on the conserved cysteine positions under different ionizing dose regimes. A verification on whether radiation threshold levels correlate to tipping points to higher oxidized species could also aid radiotherapeutics.

#### Hypothesis 2: Accumulated lamin A slows down ATM nucleoshuttling

The question now stands what consequences arise from cysteine-damaged lamin A at the nuclear envelope. Recently, C588 and C591 were additionally found to be palmitoylation sites which further inclines interest in this question from the field of cell biology (Shen and Zheng 2024).

From loss of disulfide bridges at C522, the previously discussed binding of DNA would likely get lost (Stierlé et al. 2003). Additionally, from the proposed perinuclear accumulation, a key discriminatory feature to B-type lamins gets lost when the nucleoplasmic pool of lamin A depletes (Briand et al. 2018). We can expect noticeable changes to be induced upon a decrease of nucleoplasmic lamin A, *e.g.*, proliferative changes due to growth-coregulation with LAP2α (Vidak et al. 2018).

Of interest to the RIANS model are the changes perinuclearly at the membrane. Nucleoshuttling of ATM is dependent on its passing through the nuclear pore. Lamin A does not only stabilize the envelope, but it also positions (or better say avoids) nuclear pore complexes (NPC) (Maeshima et al. 2006; Guo and Zheng 2015). Such regions of the nuclear envelope associated with lamin A are termed *pore-free islands* and they first form during mitosis, where chromosome regions far from spindle microtubules recruit B-type lamins and nucleoporins to assemble NPCs, while central regions recruit lamin A and form pore-free patches. Later, both fuse (Kwon et al. 2020). Maeshima et al. showed this phenomenon to be kept quite universally outside mitosis. Lamin A seems negatively associated with NPCs (Maeshima et al. 2006).

How this behavior is signaled, is yet controversial (Maeshima et al. 2006; Guo and Zheng 2015). If lamin A amounts are a causal reason, oxidized lamin A accumulating at the membrane could hinder nuclear pore accessibility for the shuttling of ATM. With this our hypotheses can complement the RIANS model, and more fully develop upon it.

**Fig. 12:**
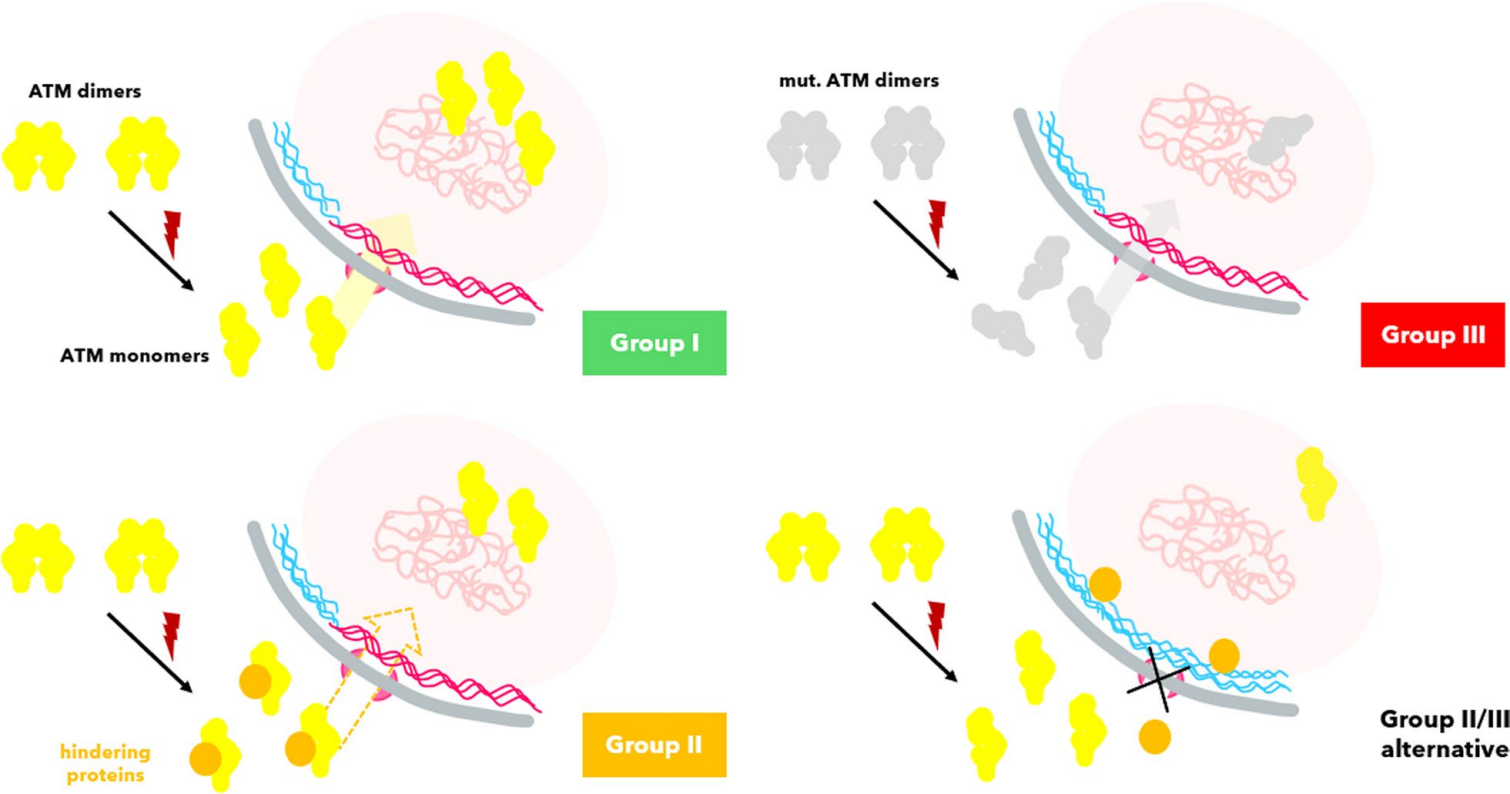
The RIANS hypothesis of ATM monomerization after ionization with Group I (normoresistant, fast translocation), Group II (moderate radiosensitivity, proposed is a blockage of the traversing ATM monomers by other mutant protein species) and Group III (highly radiosensitive, mutation in *ATM*, *LMNA*, *NBS*, *XP*, *LIG4*). Many proteins of Class III or Class II shuttle to the nucleus. It might be the case, that accumulation of oxidized lamin A (blue) at the membrane increasingly inhibits flexible nuclear pore distribution (rich regions around lamin B1, pink), thereby decreasing shuttling rates

One issue to date is that Group II individuals (5–20%) with delayed nucleoshuttling of ATM but no mutations in *ATM*, are palmed off with the explanation that a cytoplasmic pool of other mutated proteins (*e.g.* p53, Rb, neurofibromin, tuberin, huntingtin, BRCA1, BRCA2, BTK, DNMT3B, lamin A) buffers ATM’s movement towards the nucleus, illustrated in Fig. 12 (Berthel et al. 2019; Foray 2020). This may not be a full picture, as some of these proteins reside nucleoplasmically not cytosolically, or themselves shuttle between cytoplasm and nucleoplasm (*e.g.*, p53).

Additionally, lamin A does not group well with the other proteins. Progeria patients are much more radiosensitive and belong to Group III (highest radiosensitivity, lowest shuttling speed) which hints at a strong influence of accumulated progerin at the membrane on shuttling speed. From this, the accumulated lamin A form seems to not have to be functional to impair pore formation.

Foray et al. acknowledged that ATM nucleo-shuttling may not explain everything yet, and search for novel radioprotectors yielded an unexpected progeria medication (ZOLPRA) to increase nucleoshuttling (Foray 2020). We think the model has great strengths, and our hypotheses rather aids this bottleneck than dispute it. The combination of zoledronate and pravastatin (ZOLPRA) is designed to decrease an excessive accumulation of lamin A at the nuclear membrane and *Hypothesis II* thus smooth out the picture in that less accumulated lamin A allows for better nuclear pore complex distribution, speeding up ATM shuttling.

#### Hypothesis 3: Cells with higher lamin A are prone to micronuclei rupture and senescence

In genotypical cells, higher amounts of lamin A are found in stiffer tissue types where they prevent rupture of the nucleus (Srivastava et al. 2021; Swift et al. 2014). When pathogenic lamin A variants occur, or when lamin A is downregulated, or when lamin B1 is depleted, an increase of rupture is seen (Lindenboim et al. 2020).

Micronuclei, a cellular hallmark of irradiation, are predominantly surrounded by lamin A envelopes. This roots back to the nature of mitosis, where micronuclei form due to (i) lagging chromosomes, (ii) acentric fragments or (iii) anaphase bridges which recruit their own envelopes and are not integrated into the primary nucleus. As discussed above, regions close to microtubules recruit nuclear envelopes with high lamin A and few nuclear pore components while distant chromosome regions only recruit lamins B1, B2 and nucleoporins. For micronuclei, the first mechanism occurs, well reviewed by Kwon et al. (2020).

**Fig. 13:**
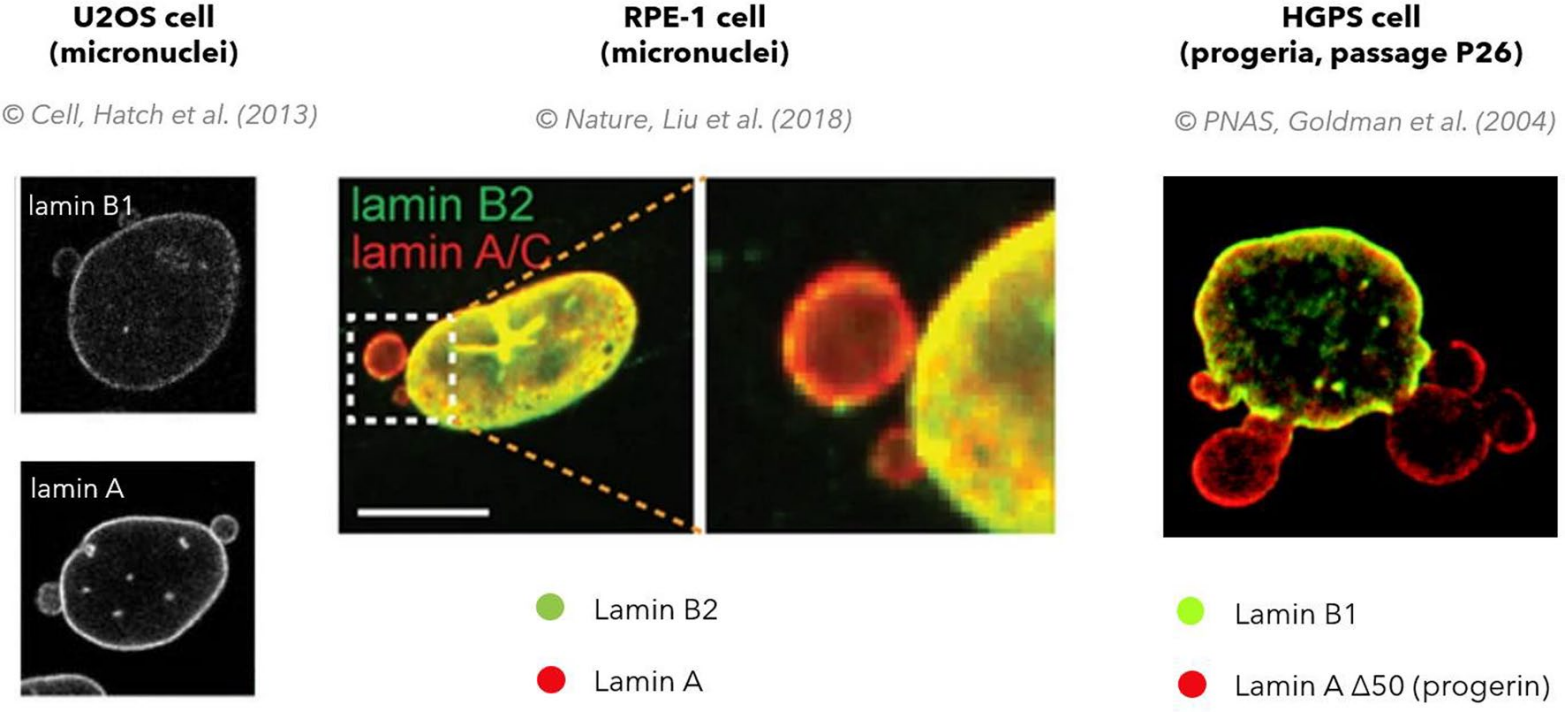
Progerin in blebs, or lamin A in micronuclei, both localize in unequal ratio to lamin B1 and B2. The nuclei shown do not depict irradiated cells. Pictures are curtesy of mentioned authors and their publishers. Hatch et al.: Reprinted from Cell, 154 (1), 47-60. Hatch EM, Fischer AH, Deernick TJ, Hetzer MW. Catastrophic Nuclear Envelope Collapse in Cancer Cell Micronuclei. Copyright (2013) with permission from Elsevier. Liu et al.: Copyright (2018) Nature, 561, 511-555. Liu S., Kwon M, Mannino M, Yang N, Renda F, Khodjakov A, Pellman D. Nuclear envelope assembly defects link mitotic errors to chromothripsis. With permission from Springer Nature. Goldman et al.: Copyright (2004) with permission from the National Academy of Sciences, U.S.A.

Resultingly, micronuclei exhibit low lamin B1 content, few nuclear pores and high levels of lamin A. Same is seen for blebs in progeria, compare Fig. 13, however, the formation of micronuclei by buddying from a dysmorphic primary nucleus is still controversial (Goldman et al. 2024; Hatch et al. 2014; Liu et al. 2018; Smith et al. 2021; Guscott et al. 2022).

In any case, the formation of micronuclei occurs as a hallmark after irradiation, creating the situation that their formation occurs in an oxidative environment. We propose lamin A becomes damaged under these circumstances (*Hypothesis I*) and Lindenboim et al. discussed how damaged lamin A predisposes for rupture, but even without that possibility, Kovacs et al. and Joo et al. demonstrated that under oxidative stress, ATR phosphorylates lamin A/C in micronuclei at serines S392, S395, further amplifying the conditions for (micro)nuclear disassembly (Lindenboim et al. 2020; Smolka and Jammerding 2023). Most recently, Di Bona et al. found another pathway of ROS-induced micronuclei destabilization via—again—oxidation of a cysteine residue in the CHMP7 protein that cascade to LEMD2, where LEMD2 is also shown to bind the lamin A C-terminus (Thanisch et al. 2017; Bona et al. 2024). Overall, micronuclei formed under oxidative stress seem fragile.

The reason for micronuclei rupture is likely not only lamin A. Many complex factors influence micronuclei clearance by autophagy or rupture (Kwon et al. 2020). High or damaged lamin A amounts could slightly predispose for rupture. When micronuclei rupture, they release endogenic DNA into the cytoplasm. This triggers cGAS-STING, a cytosolic DNA sensing pathway, which activates and nucleoshuttles IRF3, which further evokes an inflammatory response (Smolka and Jammerding 2023; Zheng 2023). Yang et al. showed that cGAS can drive cellular senescence (Yang, et al. 2017; Richter and Carine 2023). We mention this because cGAS might link micronuclei to the symptoms of inflammation or senescence in radiodegeneration, which are not yet causally elucidated.

In parallel, radiation-generated ROS can puncture mitochondria or damage the cytosolic cell membrane and activate the apoptosis caspase cascade (Rahmanian et al. 2016; Cohen-Jonathan et al. 1999). Apoptosis is the link to radiosensitivity, to the irradiation symptoms due to lethal cell death.

Apoptosis is executed by caspases, a family of cysteine-proteases, divided into initiators (caspase-2/8/9/10) and effectors (caspase-1/3/6). Micronuclei do not trigger apoptosis. However, caspases and micronuclei formation may be connected when caspases become activated to a *sub-lethal* level. At this level, caspase-3 and caspase-activated DNAse were shown to be involved in the formation of micronuclei, while caspase-6 is the only caspase capable of cleaving lamins and involved in the resolution of micronuclei (Decordier et al. 2005; Cowling and Downward 2002; Fritsch et al. 2019; Dagbay and Hardy 2017).

**We propose that the amount of ROS may influence the activation level of the caspase apoptosis cascade and lead to two regimes of non-cancerous radiation response:**

(i) high ROS: acute lethal response with apoptosis (radiosensitivity)
(ii) lower ROS: softer sub-lethal activation with micronuclei, inflammation and senescence, later genomic instability (radiodegeneration → radiosuspectibility)

This agrees with the observation by Shinomiya et al. that there exist premitotic apoptosis (before cell division) and postmitotic apoptosis response (after cell division) with different dynamics (Shinomiya et al. 2000). It also agrees with the distinction of *true radiosensitivity* where cells undergo apoptosis rapidly and die within a few hours (irrespective of mitosis and at already low DNA damage) vs. *hidden radiosensitivity* where cell death does not follow. Hidden radiosensitivities, *e.g.*, neuronal demyelination in neurons could be linked to sub-lethal caspase activity (Little 2003; Paganetti 2023).

We hypothesize that in cells with low lamin A, ROS species trigger apoptosis faster and lethally (the cells are more prone to acute radiosensitivity) while cells with higher lamin A seem to have the equivalent of an *internal buffer* that makes them more radiotolerant (but more prone to micronuclei formation and rupture, eventually running into radiodegeneration). Our initial thinking was in the other direction, that radiosensitive cells have higher lamin A levels so that ionizing radiation corrupts envelopes more. However, this thinking seems wrong, and data suggests that higher lamin A levels might act like a sulfhydryl buffer, like other radioprotectants.

When comparing Heylmann et al. (1846) who analyzed the radioresistance of different blood cell types, with Saez et al. (2020) who reviewed the lamin A/C content of immune cells, good agreement can be found. Cells with highest lamin A/C expression (dendritic cells and macrophages) were significantly the most radiotolerant. The trend was followed through other high expression, high radiotolerance types [*e.g.*, Langerhans cells (Timares et al. 2008), plasma cells (Franiak-Pietryga et al. 2022), Schwann cells (Cohen et al. 2021)].

On the other hand, radiosensitive cells, even those without high proliferation rates, show almost no lamin A expression (*e.g.*, oocytes, spermatids, cardiomyocytes, neuronal cells). This finding may aid radiotherapy in which factors cause apoptosis. Currently, proliferation rate is the measurement to gauge radiosensitivity of tissues, and radiosensitivity of these cell types cannot be explained with their (low) proliferation rate. Figure 15 (next page) compares the expression of *LMNA*, *LMNB1*, *LMNB2* for non-cancer cell types.

**Fig. 14:**
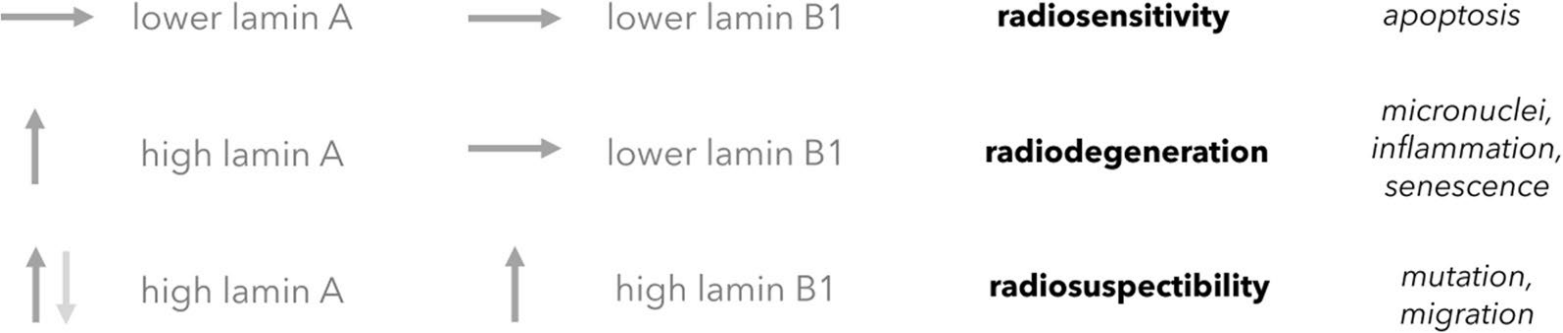
Summary of *Hypothesis III* and *IV*. Low lamin A and low lamin B1 do not support ROS-buffering and lead to lethal apoptosis activation (first row, radiosensitivity). If a cell-internal sulfhydryl buffer, *e.g.* high lamin A, can quench the apoptotic cascade to below sub-lethal threshold, the effects of micronuclei and inflammation, as well as progeria-like senescence will eventually occur (second row, radiodegeneration). Low levels of B-type lamins aid a senescent phenotype. However, higher levels of lamin B1 (third row, radiosuspectibility) can activate a better ROS-response and may reinduce proliferation, as well as cell migration, at the cost of an instable genome

**Fig. 15:**
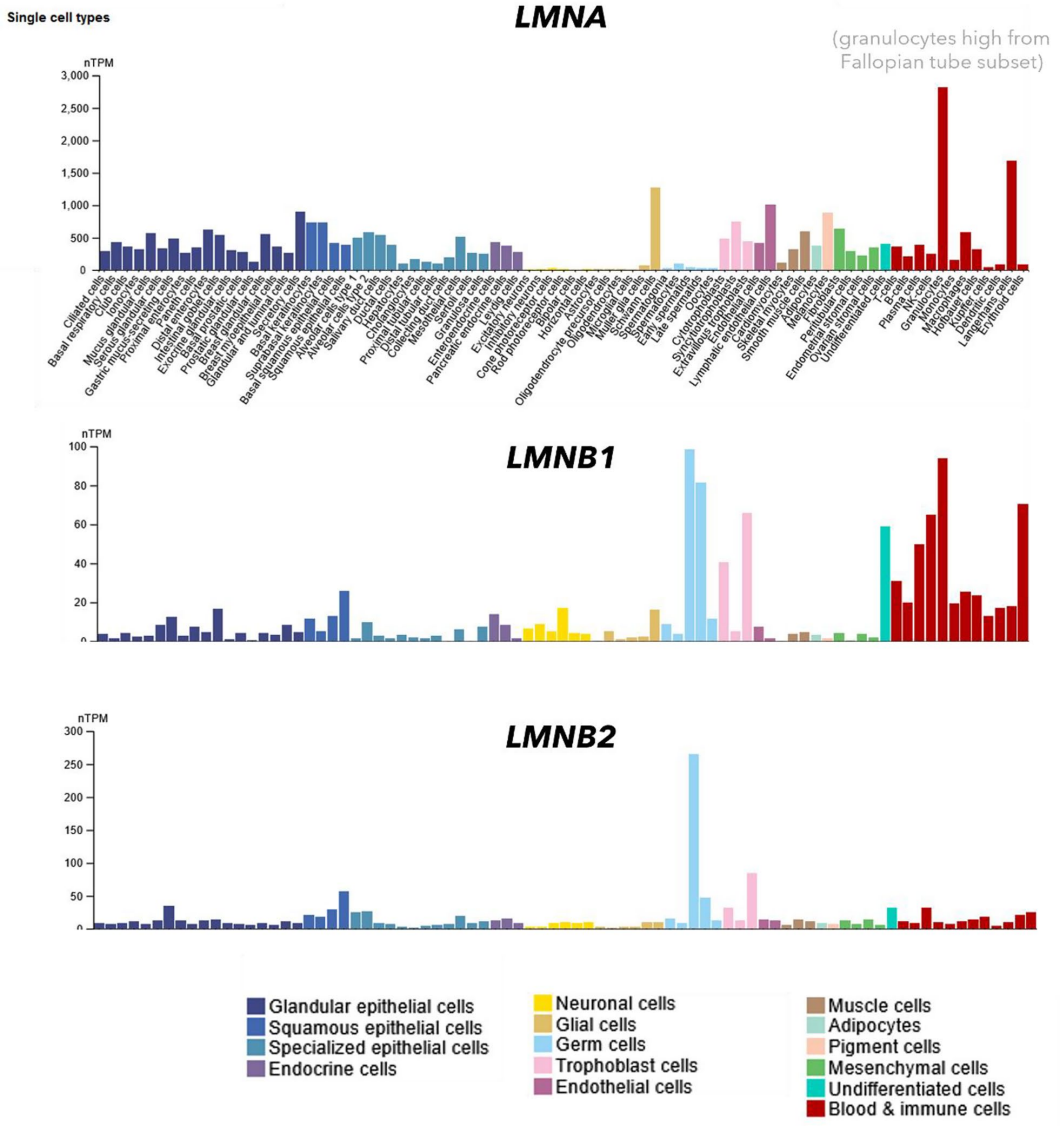
Levels of *LMNA/C*, *LMNB1*, *LMNB2* in healthy cells. The scaling of the different axes shows a much higher expression of lamin A/C over B-type lamins. Human Protein Atlas (2025a, b, c)

We summarize *Hypothesis III* and *IV* in Fig. 14. *Hypothesis IV* continues in the following section. *Hypothesis III* is our most uncertain one, the pool of lamin A may be quite too small (plasma level: 6.7 µg/L (Lamin and protein concentration plasma 2025), cells internally: Fig. 15) and other protein levels unique per cell types could match the above explained agreements instead of our hypothesis.

#### Hypothesis 4: Cells with higher lamin B1 can compensate towards stochastic response

If cells avoid acute radiosensitivity, they (i) may enter a senescent state or (ii) proliferate. In both cases, their genomes are left in a vulnerable state and repair will be essential. With the proposal of damaged lamin A, the envelope and repair machinery would be additionally impaired.

While functional lamin A stiffens the nuclear envelope, B-type lamins can allow it to be more malleable. Smith et al. showed that B-type lamins can compensate nuclear abnormalities in presence of mutant lamin A, other functions of B-type lamins are the interaction with nuclear pores (discussed in *Hypothesis II*), and the support of migration of cells through confined spaces due to more flexible nuclear mechanoproperties (Pekovic et al. 2011; Etourneaud et al. 2021; Lv et al. 2024a; Smith et al. 2021). Malhas et al. demonstrated that lamin B1 is additionally a key activator of the oxidative stress response via Oct-1 (Paul and Fulka 2022).

In Ataxia telangiectasia patients and in chronic kidney disease, an ATM-independent lamin B1 upregulation in response to oxidative stress was found. The pathway causes nuclear alterations and eventually leads to senescence via p38 and MAPK (Barascu et al. 2012; Wang et al. 2020).

Normally, high expression of lamin B1 and B2 is seen in embryogenesis and proliferative development, lamin A dominates later during differentiation (Vergnes et al. 2004). A recent review underlines the diverse picture the scientific community currently holds on lamin B1 (Lv et al. 2024b). In contrast to what was found for ataxia telangiectasia, it is mostly accepted that low lamin B1 is a senescence biomarker, *e.g.*, Dreesen et al. on how lamin B1 declines with senescence (Dreesen et al. 2013), and Freund et al. on how if irradiation decreases lamin B1, a senescent phenotype follows (Freund et al. 2012; Kristiani and Kim 2023).

**Fig. 16:**
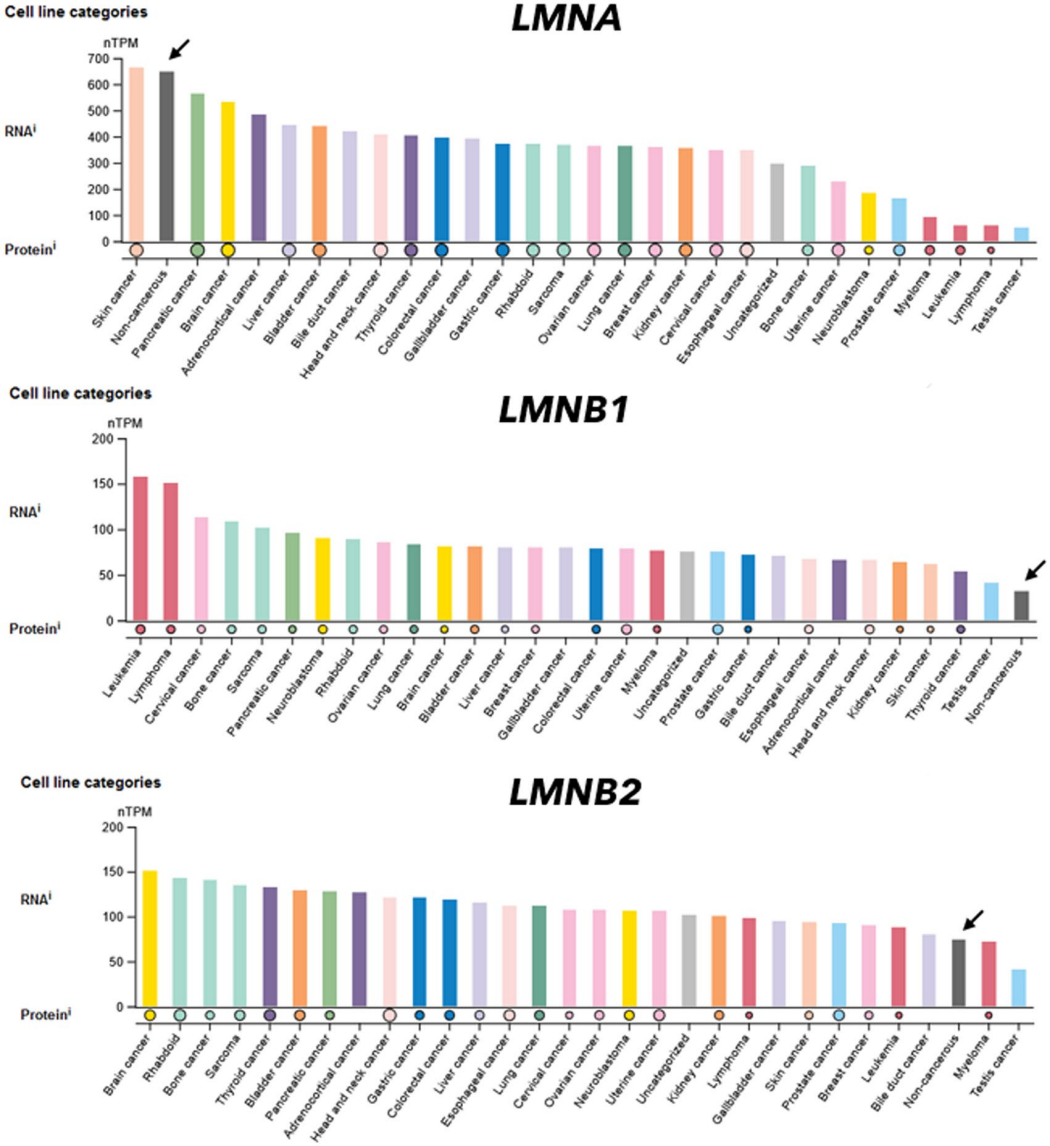
Altered levels of lamins in cancer cell lines. Protein levels as circle below the bar plots. The comparator dataset of non-cancerous cells is displayed as black bar and indicated with a black error. All bars are sorted from highest to lowest mRNA expression level. Colors follow the color legend of Fig. 16. *Human Protein Atlas* (2025a, b, c)

On the other end of the spectrumlie high levels of lamin B1, accepted as proliferation marker, compare Fig. 16. Every cancer cell line listed in the *Human Protein Atlas* has above-normal lamin B1 expression, (normal tissue indicated with black arrows, for *LMNA* the opposite holds true) (Human Protein Atlas (PDB 2025).

What Malhas et al. (Paul and Fulka 2022) suggest is that lamin B1 upregulation can be a response to oxidative stress response and can improve survival, however, in irradiated cell, this would come at the cost of moving away from senescence—moving forward with an instable genome.

This would explain the eventual onsets of cancer, of radiosusceptibility. Our hypothesis is that lamin B1 comes to sit at the fork of two contradictory molecular pathways between senescence and cell cycle continuation: If an irradiated cell transforms into a state of lamin B1 upregulation, it can better handle ROS, which may sufficiently equip it to circumvent senescence pathways and stay in the cell cycle.

Cell populations would carry on with damaged DNA, an envelope with increased lamin B1 and increased malleability as well as migration potential, and if *Hypothesis I* holds true, at impaired lamin A levels with possibly instable DNA repair response machinery.

**Fig. 17:**
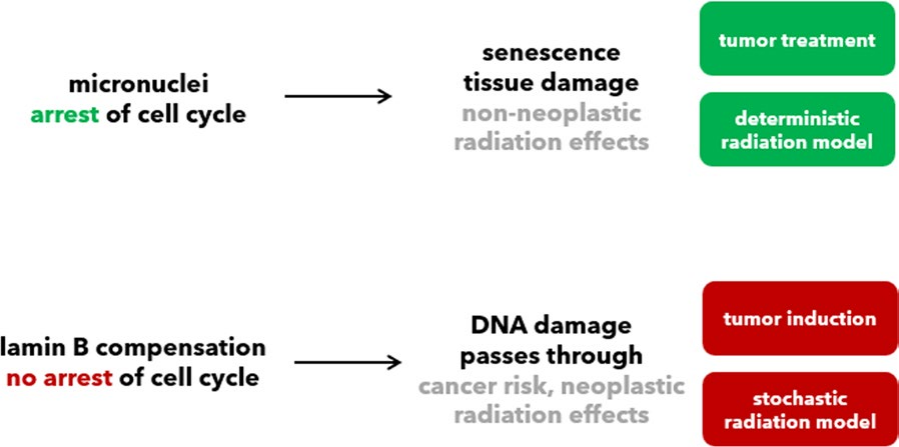
Deterministic (green, top) and stochastic response (red, bottom) in the context of lamins. A “buffer” of lamin A unique per cell type might define a radiosensitivity threshold and control apoptosis or senescence. For neoplastic response, the known mechanism is of cells prevailing with DNA damage, but at insufficient repair levels. This could be the case, if lamin A is damaged but lamin B overexpressed, circumventing senescence

The migration ability lamin B1 brings to nucleus, may also be the case why *LMNB1* and *LMNB2* are upregulated in cancer, while stiffening *LMNA* expression reduces, see Fig. 16 (Dreesen et al. 2013). A scenario after irradiation where dysfunctional lamin A and upregulated lamin B1 are present would exactly mirror this situation. The multiple transformation steps from irradiated cell to a (secondary) tumor could further downregulate functional lamin A again (light gray arrow, Fig. 15). In both cases, higher metastatic potential for cancers from radiation origin may arise (Fig. 17).

#### Hypothesis 5: Radiosensitivity of dividing cells due to exposed lamins

Lastly, we discuss the phosphorylation of lamins which is required for mitotic execution, and how damaged lamins might affect the cell cycle via nuclear assembly and the cell cycle radiosensitivity.

Lamins have various phosphorylation sites, more than 70 phosphorylation sites are identified across lamins, 20 in lamin A alone (Machowska et al. 2015). When lamin A is phosphorylated outside of mitosis, it detaches from the envelope and takes position to operate inside the nucleoplasm (Liu et al. 2025). During mitosis, all lamins are phosphorylated by Cdk1 at mitotic sites (for lamin A p.Ser22, p.Ser392, p.Ser394) which allows for complete disassembly of the nucleus. For reassembly, these sites are dephosphorylated again and mitosis can be exited (Machowska et al. 2015; Liu et al. 2025; Torvaldson et al. 2015).

**Fig. 18:**
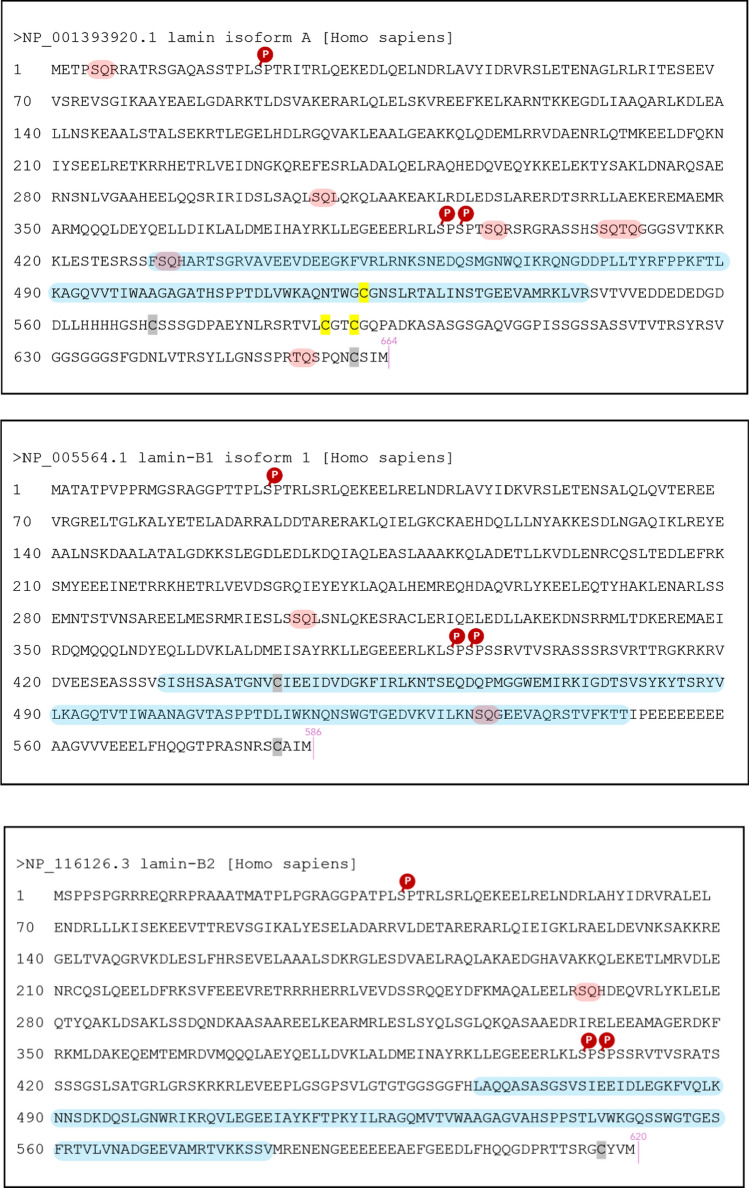
Display of the *Homo sapiens* lamins A, B1, B2. The phosphorylation sites of Cdk1 near the coiled rod domains for mitotic disassembly are marked (red, white -P). The Ig-fold is highlighted in blue. Possible SQ/TQ motives for ATR or ATM are overlayed in red. Lamin A has seven possible SQ/TQ phosphorylation sites

Figure 18 shows an overview of lamin A, lamin B1 and B2 with the key mitotic sites.

During analysis of our sequences, lamin A was found to hold *SQ/TQ motifs*. This strengthens the notion to think about lamin A in the family of radiation response proteins, as SQ/TQ motifs are preferential phosphorylation sites for ATM and ATR and used to screen DNA repair proteins (Traven and Heierhorst 2005; Cara et al. 2014). For ATM no data is available, but ATR can phosphorylate lamin A at p.Ser282, p.Ser392, p.Ser395 (Kovacs et al. 2023; Joo et al. 2023). Lamin B1 and B2 hold much fewer such motifs. This justified our focus for radiobiologists on lamin A from another perspective to progeria similarities.

### Phosphorylation during mitosis

**Fig. 19:**
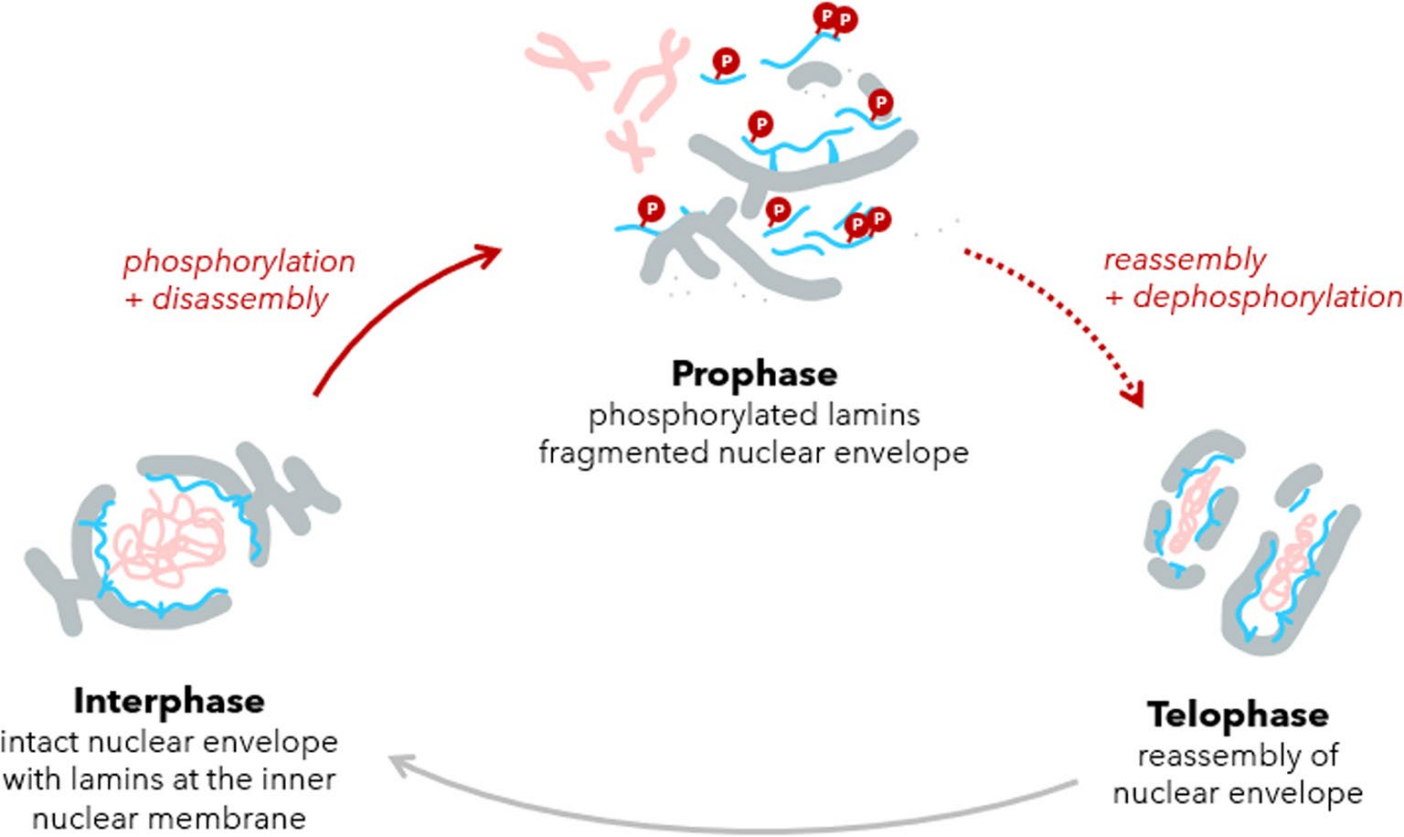
Phosphorylation of lamins (blue). Disassembly and reassembly of the nuclear envelope (grey) is necessary for mitosis. The successful (de-)phosphorylation is tightly linked to correct completion of the cell cycle. During phosphorylation, the exposed lamins are highly prone to oxidation and radiation damage, drawing a novel picture of the high radiosensitivity in highly proliferative tissues. Picture adapted after Alberts (Alberts 2015)

In the context of the cell cycle, phosphorylation of lamins opens the question of whether damaged lamin A would allow for correct disassembly and reassembly of the nuclear envelope (see Fig. 19). Would cells be able to enter, execute, exit mitosis? We suggest that if *Hypothesis I* holds true, this might not be the case.

We propose a slight perspective shift with additional explanation for why rapidly dividing tissues are highly radiosensitive. The standard explanation is that during the cell cycle, DNA is replicated in *S* phase and more divisions lead to more error accumulation, eventually lethal. This principle is utilized in radiotherapy, where highly proliferating tissue (= cancer) is preferentially killed by radiation. Interestingly, the most radiosensitive cell cycle phases seem sometimes the *S* phase (Otani et al. 2016), where the chromatin is most exposed, but more often the late *G2/M* phase and the mitotic *M* phase (Dietmar and Keng 1984; Blakely et al. 1989; Hill et al. 2023; Nakayama et al. 2011; Syljuåsen 2019).

Hill et al. and Zhao et al. demonstrated that apart from apoptosis, an accumulation of cells in the *G2/M* phase after irradiation occurs. The accumulation starts after 6 h and levels off after 30 h for doses above 6 Gy (Hill et al. 2023; Zhao et al. 2018). This time window suggest that likely the initially irradiated cell, not a daughter cell, is impaired.

Without a doubt, inhibitors of checkpoint kinases (especially for *G2/M*) have advanced cancer treatment in the recent years, which were built onto the notion that accumulated DNA damage kills cells when a checkpoint cannot be passed. However, this susceptible *G2/M* transition is also defined by the critical step of nuclear lamina disassembly. The mechanobiology of the nuclear envelope at this *G2/M* transition is excellently reviewed by Lima et al. (Lima and Ferreira 2024).

The phosphorylation of lamins which has to allow the disassembly, mitotic sites illustrated in Fig. 18, is executed by Cdk1, and a range of cell cycle kinases are responsible for lamin phosphorylations, *e.g.* Cdk5, PKC, AKT, ATR, GSK3β, CK2, BUBR1 (Torvaldson et al. 2015; Kovacs et al. 2023; Kochin and Shimi 2014; Ying et al. 2023; Zhang et al. 2023).

Checkpoint kinase inhibitors might have a direct effect on lamin disassembly and the effectiveness of checkpoint inhibitors could partially stem from this aspect. Unsuccessful phosphorylation of lamins and lack of disassembly might halt cells at the *G2/M* checkpoint, as unsuccessful reassembly might forbid the exit from the *M* phase.

We propose the following two attributes to impact cells in *G2/M* and *M* after irradiation:

- First, the experimentally observed accumulation of cells in the *G2/M* phase after irradiation may be explained by irradiated cells arriving with damaged lamin at the *G2/M* transition but not being able to trigger a correct disassembly.
- Second, the high intrinsic radiosensitivity of late *G2/M* and *M* phases may be due to disassembled lamins being fragile targets due to (i) lack of stabilization from the envelope to dissipate energy, and (ii) heightened exposition to cytosolic ROS species.

**Fig. 20:**
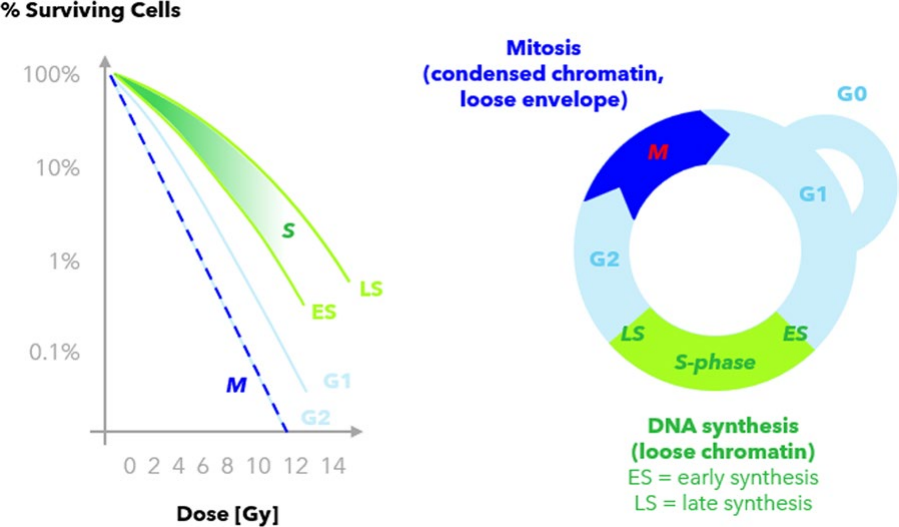
Radiosensitivity of cell cycle phases (Hill et al. 2023; Syljuåsen 2019; Universität Bern 2015). The surviving cell fractions report on Chinese hamster cells (left, logarithmic y-axis). Mammalian cells exposed to ionizing radiation are most sensitive in the M phase (blue) and least sensitive in the synthesis phase (green) (right, cell cycle stages). Nuclear de- and reassembly, as well as the G2/M checkpoint for DNA damage, define success or apoptosis

Together, we suggest that lamins, specifically lamin A, might be a significant attributor to the radiosensitivity of proliferating tissues at these stages (Fig. 20). While DNA occurs in its most condensed form as chromosomes, the exposure of lamins is most increased in *G2/M* and *M*.

Finally, we asked the questions if phosphorylated sites could add an additional vulnerability to radiation to a protein. The rationale would be that heavier atomic species like sulfur or phosphor mark radiation absorption hotspots in biomolecules, with larger photoabsorption cross section, but only for X-rays (Erk 2013). In the case of secondary damage by ROS species, or ionizing particles which comprise the prevalent space radiation hazard, this is not applicable.

### Open definitions on radiosensitivity

We hope to have encouraged the notion of radiosensitivity and radiodegeneration in the context of the nuclear envelope. We are aware that there are many open definitions in the field of radiobiology, on (i) how to classify radiation symptoms, (ii) how to measure sensitivity and (iii) how to predict patient outcomes.

The question is dual, whether there are accurate predictors on the cellular level before irradiation to identify vulnerable individuals, and in the clinics for prognostics and early, targeted intervention, and this for all three risk categories: radiosensitivity, radiodegeneration and radiosuspectibility.

Baria et al. found early a link between *G2* lymphocyte radiosensitivity and breast/colon cancer patients (genetic background) which is absent in lung/cervix cancer (environmental aetiology) (Baria et al. 2001). This showed that cellular response may correlate with patient risk, however, ionizing radiation being an environmental carcinogen, the non-genetic risk has to be measured.

Pantelias and Terzoudi et al. describe a standardized way to measure *G2* cell cycle phase sensitivity of lymphocytes to ionizing irradiation by applying a correction against a caffeine-*G2/M*-checkpoint-suppressed culture to assimilate the cellular state of ataxia telangiectasia patients and make the assay comparable for such genetic backgrounds (Pantelias and Terzoudi 2011; Terzoudi et al. 2009).

Ways to evaluate such cell assays on radiosensitivity are (i) to measure the surviving fraction after irradiation (*e.g.* after 2 Gy SF2%), (ii) to count genetic damage via γH2AX repair foci assays with immunofluorescent methods, or (iii) micronuclei via comet assay.

Results do not always translate to patient radiosensitivity. Interpersonal and intrapersonal variation on the assays hinder assessments. The surviving fraction is exemplarily strongly dependent on cell phase (*Hypothesis V*), cell type, and genotype. This was one reason why Joubert et al. classified fibroblasts of eight genetic backgrounds comparatively and found four classes of behavior after irradiation (2 Gy) which they analyzed in context of three radiation response proteins ATM, MRE11 and DNA-PK (Joubert et al. 2007).

Towards a theoretical definition, Bodgi et al. proposed a formula that can fit NBS1, pATR, 53BP1, BRCA1 and MRE11 protein dynamics at repair foci. The Bodgi formula is constructed upon γH2AX foci appearance time and γH2AX disappearance time. When fitting patient data, they could identify three different groups of radiosensitivity with three different rates of ATM shuttling to the nucleus (Bodgi et al. 2013). Upon this, the RIANS model was built (Berthel et al. 2019).

A standardization project to measure radiosensitivity (Copernic project, established in 2004) uses irradiation of fibroblasts at 2 Gy and measurement of γH2AX, MRE11, pATM foci after 10 min., 1 h, 4 h, 24 h. Copernic is designed after the RIANS model and in the most recent evaluation in 2024, they showed that the currently most representative biomarker for radiosensitivity is the maximal number of pATM foci. Not spontaneous or residual micronuclei level, nor residual γH2AX foci, can reliably predict CTCAE grades (Foray 2020; Sonzogni et al. 2024).

On genetics, El-Nachef et al. list a table of genes that can impact SF2%: *ATM*, *LIG4*, *NBS1*, *LMNA*, *BTK*, *LIGI*, *DNMT3B*, *GSS*, *RAD50*, *MRE11*, *CS/XP*, *USH*, *HTT*, *DMD*, *FANCA*, *BLM*, *PTCH1*, *TSC*, *NF1*, *p53*, *APC*, *MLH*, *RB1*, *BRCA*, *WRN*, *RecQL4*, *NHEJ1*, *DCLRE1C*, *MLH1*, *MSH2/6* (El-Nachef et al. 2021). Lamin A with progeria is to date the fourth most radiosensitive genetic syndrome listed, indicating that patients with lamin A C-terminally impaired struggle with radiation response.

## Discussions and conclusions

We analyzed radiation effects for astronauts in space and were confronted with transient laminopathy-like symptoms. Starting with known classes of radioprotectors, we identified endogenous radiation targets distinct from DNA, specifically three conserved cysteines in the lamin A C-terminus (C522, C588 and C591). The sites establish disulfide bonds and engagement with chromatin and proteins in the nucleoplasm.

The molecular “injury” of a *space* or *radiation laminopathy* would proceed via oxidation. Generally, radiation takes primary or secondary routes to cause cellular damage. Direct primary radiation damage is initiated within femtoseconds and breaks O–H, N–H, S–H and C-H bonds. Examples are particles directly hitting DNA, or the O–H bond in water being broken to rapidly generate reactive oxygen species (ROS). In a secondary cascade, these ROS further oxidize proteins.

To date, the nucleus is mainly studied under primary radiation damage, direct DNA breaks. How secondary radiation effects (*e.g.* ROS from water radiolysis) affect other nuclear components, like the lamins, is unclear.

We manually evaluated 11,300 abstracts on *in-vitro*, *in-vivo*, 1G-cohorts (cancer), exposed medical and military workers, and astronaut which were curated on radiobiology for the ESA Systematic Analysis of Threats in Space (STARS project) and not one abstract directly showed data on lamins, while nuclear deformity and micronuclei are abundantly discussed. However, genomic integrity is determined both by nuclear content and its envelope.

We suggest a novel mechanistic working hypothesis: Radiation may oxidize the lamin A C-terminus, causing a loss of -SH and -S–S- groups at large doses, and loss of binding to DNA. The oxidized lamin A would relocate to a perinuclear state, restructuring the nuclear membrane.

As clinically relevant points, we suggest that the level of lamin A determines a threshold resistance capability per cell type. In cells with high lamin A, more reactive oxygen species can be scavenged, making them more radioresistant but eventually victims to senescence and radiodegeneration, possibly via sub-lethal caspase activation or cGAS-STING activation.

In cells low lamin A, the apoptotic cascade executes fully at start, causing radiosensitivity.

By integrating B-type lamins into the picture, neoplastic response can be understood. High levels of lamin B1 are correlated with (cancer) proliferation, at the same time lamin B1 can activate oxidative stress responders. Under ROS exposure, lamin B1 overexpression is induced. By elevating lamin B1 levels, cells survive oxidative stress better and can stay in the cell cycle, however, at the cost of instable genomes projecting cancer risk into following generations.

A vulnerability of lamins would additionally suggest a novel picture for why highly proliferative tissue is radiosensitive. During mitosis, the nuclear lamina disassembles. Phosphorylation and dephosphorylation of lamins regulates the process, however, in their disassembled state, lamins become highly exposed. Together, not only the *G2/M* checkpoint on DNA damage but also on lamin phosphorylation, and exposed lamins over the whole mitotic stage, may make proliferating tissue highly radiosensitive.

These hypothesis opens new perspectives with interesting questions. We hope to see more radiation research on the nuclear envelope. A rational design of new radioprotector classes would be a trailblazing step for oncology and—if treatments can be transferred—laminopathy care. Already today, radioprotectors.org overlaps with geroprotectors.org.

With care, the mechanistic model could be tested per hypothesis. Long-duration spaceflight, as well as patients undergoing radiotherapy and new radiotherapeutics, could benefit from an understanding of whether and how the nuclear membrane is impacted by ionizing radiation.

## Supplementary Information

Supplementary material 1.

## Data Availability

No datasets were generated or analysed during the current study.

## Footnotes

1 ^*^ Li Fraumeni’s syndrome: radiosensitivity (tissue) → , radiosuspectibility (cancers) ↑. Cockayne’s syndrome: radiosensitivity (tissue) ↑, radiosuspectibility →. Ataxia telangiectasia: radiosensitivity (tissue) ↑, radiosuspectibility (cancers) ↑.
