# Supplementary material for: Radiation symptoms resemble laminopathies and the physical underlying cause may sit at the lamin A C-terminus
Source: Mol Med. 2025 Feb 20;31:69. doi: 10.1186/s10020-025-01081-0 (PMC11844092; doi:10.1186/s10020-025-01081-0)
Supplement: Supplementary file 1 — Supplementary material 1. [file 10020_2025_1081_MOESM1_ESM.docx]

**References in Supplementary Information**

R1. DiCarlo, A. L., Maher, C., Hick, J. L., Hanfling, D., Dainiak, N., Chao, N., Bader, J. L., Coleman, C. N. and Weinstock, D. M. (2011). Radiation Injury After a Nuclear Detonation: Medical Consequences and the Need for Scarce Resources Allocation. *Disaster Med Public Health Prep*. **5** (S1) pp. S32-S44. <https://doi.org/10.1001/dmp.2011.17>.

R2. Carr, S. M. The millisievert and milligray as measures of radiation dose and exposure. <https://www.mun.ca/biology/scarr/Radiation_definitions.html>.

R3. United States Nuclear Regulatory Commission. Lethal dose (LD). <https://www.nrc.gov/reading-rm/basic-ref/glossary/lethal-dose-ld.html>.

R4. European Nuclear Society. Lethal dose. <https://www.euronuclear.org/glossary/lethal-dose/>

**Supplementary Information**

**Figure S1**

Figure S1: Multiple sequence alignment (ClustalW2, online via: <https://www.ebi.ac.uk/jdispatcher/msa/clustalo>) of lamin A protein sequences from differently radiosensitive species, submitted FASTA sequences according to Table S2. The Mongolian gebril has a mutation at the Ig-fold in context from the conserved C522 (Homo sapiens) to **threonine** (yellow, top panel). The mutation is of loss-of-function character in the context of disulfide bridging, however, a gain-of-function in the context of ROS resistance. Another mutation from **T590** to **alanine** occurred (yellow, bottom panel) between the C588 and C591 cysteines. This residue is not a known phosphorylation site^111^ which would get lost, thereby the mutation is likely less influential.


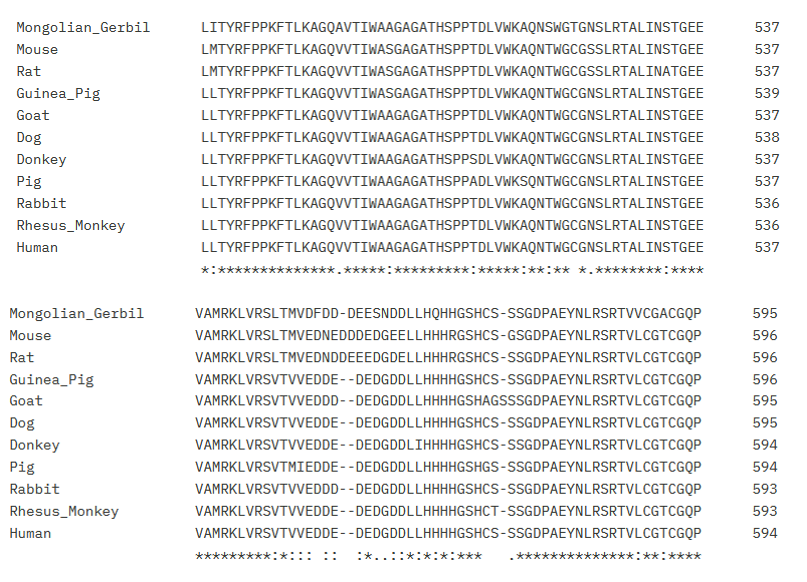


**Table S1 (reproduced, reviewed from^R1^)**

Table S1: Ten mammal species and *Homo sapiens* with representative radiation sensitivity, measured by lethal dosis for 50% of the population after an exposure for 30 days (LD_50/30_). Sorted from highest to lowest radiation sensitivity (top to bottom).

| **SPECIES** | **LD_50/30_ [cGy]** | **Scientific Name** |
| --- | --- | --- |
| Mongolian gerbil | 1000 | *Meriones unguiculatus* |
| Rabbit | 750 | *Oryctolagus cuniculus,* |
| Rat | 714 | *Rattus norvegicus* |
| Mouse | 640 | *Mus musculus* |
| Rhesus monkey | 600 | *Macaca mulatta* |
| Human (added from^R2/R3/R4^) | 300-500 | *Homo sapiens* |
| Guinea pig | 450 | *Cavia porcellus* |
| Donkey | 255 | *Equus asinus* |
| Dog | 250 | *Canis lupus familiaris* |
| Pig | 250 | *Sus scrofa* |
| Goat | 240 | *Capra hicus* |

**Figure S2 – Lamin A (MSA, Phylogenetic tree)**

Figure S2: Result on phylogenetic distances between the species determined strictly from lamin A protein multiple sequence alignment (Clustal Omega 2, inputs see Table S2). The phylogenetic ordering mimics the order of radiation sensitivity for the species respectively (compare Table S1).


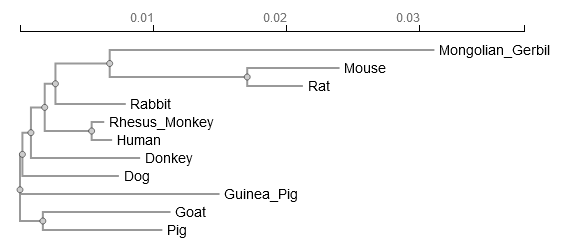


**Table S2 – Lamin A**

Table S2: Input NCBI accession numbers and FASTA amino acid sequences for multiple sequence alignment between ten mammal orthologs and the human lamin A protein. “aa” = Amino acid, the length of sequence per ortholog is stated left to the FASTA. Results see Figure S1 and Figure S2.

| **SPECIES** | **NCBI Accession Reference** |  | **FASTA Lamin A ortholog** |
| --- | --- | --- | --- |
| **Mongolian gerbil**  *Meriones unguiculatus* | <https://www.ncbi.nlm.nih.gov/protein/XP_021493399.1> | 664 aa | >XP_021493399.1 lamin [Meriones unguiculatus]  METPSQRRTTRSGAQASSTPLSPTRITRLQEKEDLQELNDRLAVYIDRVRSLETENAGLRLRITESEEVV  SREVSGIKAAYEAELGDARKTLDSVAKERARLQLELSKVREEFKELKARNTKKEGDLLAAQARLKDLEAL  LNSKEAALSTALSEKRTLEGELHDLRGQVAKLEAALGEAKKQLQDEMLRRVDAENRLQTLKEELDFQKNI  YSEELRETKRRHETRLVEIDNGKQREFESRLADALQELRAQHEDQVEQYKKELEKTYSAKLDNARQSAER  NSNLVGAAHEELQQSRIRIDSLSAQLSQLQKQLAAKEAKLRDLEDSLARERDTSRRLLAEKEREMAEMRA  RMQQQLDEYQELLDIKLALDMEIHAYRKLLEGEEERLRLSPSPTSQRSRGRTSSHSSQSQGAGSTTKKRK  LESTESRSSFSQHARTSGRVAVEEVDEEGKFVRLRNKSSEDQSMGNWQIKRQNGDDPLITYRFPPKFTLK  AGQAVTIWAAGAGATHSPPTDLVWKAQNSWGTGNSLRTALINSTGEEVAMRKLVRSLTMVDFDDDEESND  DLLHQHHGSHCSSSGDPAEYNLRSRTVVCGACGQPADKAAGGSGAQAGGSVSSGSSASSVTVTRSYRSVG  GSGGGSFGDSLVTRSYLLGSSSPRTQSPQNCSIM |
| **Rabbit**  *Oryctolagus cuniculus* | <https://www.ncbi.nlm.nih.gov/protein/XP_008262547.1> | 663 aa | >XP_008262547.1 lamin isoform X1 [Oryctolagus cuniculus]  METPSQRRATRSGAQASSTPLSPTRITRLQEKEDLQELNDRLAVYIDRVRSLETENAGLRLRITESEEVV  SREVSGIKAAYEAELGDARKTLDSVAKERARLQLELSKVREEFKELKARNTKKEGDLMAAQARLKDLEAL  LNSKEAALSTALSEKRTLEGELHDLRGQVAKLEAALGEAKKQLQDEMLRRVDAENRLQTLKEELDFQKNI  YSEELRETKRRHETRLVEIDNGKQREFESRLADALQELRAQHEDQVEQYKKELEKTYSAKLDNARQSAER  NSNLVGAAHEELQQSRIRIDSLSAQLSQLQKQLAAKEAKLRDLEDSLARERDTSRRLLAEKEREMAEMRA  RMQQQLDEYQELLDIKLALDMEIHAYRKLLEGEEERLRLSPSPTSQRRGRASSHSSQTQGGGSITKKRKL  ESAESRSSFSQHARTSGRVAVEEVDEEGKFVRLRNKSNEDQSMGNWQIKRQNGDDPLLTYRFPPKFTLKA  GQVVTIWAAGAGATHSPPTDLVWKAQNTWGCGNSLRTALINSTGEEVAMRKLVRSVTVVEDDDDEDGDDL  LHHHHGSHCSSSGDPAEYNLRSRTVLCGTCGQPADKASAGGPGAQVGGSISSGSSASSVTVTRSYRSVGG  SGGGSFGDNLVTRSYLLGNSSPRTQSPQNCRIM |
| **Rat**  *Rattus norvegicus* | <https://www.ncbi.nlm.nih.gov/protein/NP_001002016.2> | 665 aa | >NP_001002016.2 prelamin-A/C [Rattus norvegicus]  METPSQRRATRSGAQASSTPLSPTRITRLQEKEDLQELNDRLAVYIDRVRSLETENAGLRLRITESEEVV  SREVSGIKAAYEAELGDARKTLDSVAKERARLQLELSKVREEFKELKARNTKKEGDLLAAQARLKDLEAL  LNSKEAALSTALSEKRTLEGELHDLRGQVAKLEAALGEAKKQLQDEMLRRVDAENRLQTLKEELDFQKNI  YSEELRETKRRHETRLVEIDNGKQREFESRLADALQELRAQHEDQVEQYKKELEKTYSAKLDNARQSAER  NSNLVGAAHEELQQSRIRIDSLSAQLSQLQKQLAAKEAKLRDLEDSLARERDTSRRLLAEKEREMAEMRA  RMQQQLDEYQELLDIKLALDMEIHAYRKLLEGEEERLRLSPSPTSQRSRGRASSHSSQSQGGGSVTKKRK  LESSESRSSFSQHARTSGRVAVEEVDEEGKFVRLRNKSNEDQSMGNWQIRRQNGDDPLMTYRFPPKFTLK  AGQVVTIWASGAGATHSPPTDLVWKAQNTWGCGSSLRTALINATGEEVAMRKLVRSLTMVEDNDDEEEDG  DELLHHHRGSHCSSSGDPAEYNLRSRTVLCGTCGQPADKAASGSGAQVGGSISSGSSASSVTVTRSFRSV  GGSGGGSFGDNLVTRSYLLGNSSPRTQSSQNCSIM |
| **Mouse**  *Mus musculus* | <https://www.ncbi.nlm.nih.gov/protein/NP_001002011.2> | 665 aa | >NP_001002011.2 prelamin-A/C isoform A precursor [Mus musculus]  METPSQRRATRSGAQASSTPLSPTRITRLQEKEDLQELNDRLAVYIDRVRSLETENAGLRLRITESEEVV  SREVSGIKAAYEAELGDARKTLDSVAKERARLQLELSKVREEFKELKARNTKKEGDLLAAQARLKDLEAL  LNSKEAALSTALSEKRTLEGELHDLRGQVAKLEAALGEAKKQLQDEMLRRVDAENRLQTLKEELDFQKNI  YSEELRETKRRHETRLVEIDNGKQREFESRLADALQELRAQHEDQVEQYKKELEKTYSAKLDNARQSAER  NSNLVGAAHEELQQSRIRIDSLSAQLSQLQKQLAAKEAKLRDLEDSLARERDTSRRLLAEKEREMAEMRA  RMQQQLDEYQELLDIKLALDMEIHAYRKLLEGEEERLRLSPSPTSQRSRGRASSHSSQSQGGGSVTKKRK  LESSESRSSFSQHARTSGRVAVEEVDEEGKFVRLRNKSNEDQSMGNWQIRRQNGDDPLMTYRFPPKFTLK  AGQVVTIWASGAGATHSPPTDLVWKAQNTWGCGSSLRTALINSTGEEVAMRKLVRSLTMVEDNEDDDEDG  EELLHHHRGSHCSGSGDPAEYNLRSRTVLCGTCGQPADKAAGGAGAQVGGSISSGSSASSVTVTRSFRSV  GGSGGGSFGDNLVTRSYLLGNSSPRSQSSQNCSIM |
| **Rhesus monkey**  *Macaca mulatta* | <https://www.ncbi.nlm.nih.gov/protein/NP_001244822.1> | 663 aa | >NP_001244822.1 lamin [Macaca mulatta]  METPSQRRATRSGAQASSTPLSPTRITRLQEKEDLQELNDRLAVYIDRVRSLETENAGLRLRITESEEVV  SREVSGIKAAYEAELGDARKTLDSVAKERARLQLELSKVREEFKELKARNTKKEGDLMAAQARLKDLEAL  LNSKEAALSTALSEKRTLEGELHDLRGQVAKLEAALGEAKKQLQDEMLRRVDAENRLQTMKEELDFQKNI  YSEELRETKRRHETRLVEIDNGKQREFESRLADALQELRAQHEDQVEQYKKELEKTYSAKLDNARQSAER  NSNLVGAAHEELQQSRIRIDSLSAQLSQLQKQLAAKEAKLRDLEDSLARERDTSRRLLAEKEREMAEMRA  RMQQQLDEYQELLDIKLALDMEIHAYRKLLEGEEERLRLSPSPTSQRSRGRASSHSSQTQGGGSVTKKRK  LESESRSSFSQHARTSGRVAVEEVDEEGKFVRLRNKSNEDQSMGNWQIKRQNGDDPLLTYRFPPKFTLKA  GQVVTIWAAGAGATHSPPTDLVWKAQNTWGCGNSLRTALINSTGEEVAMRKLVRSVTVVEDDEDEDGDDL  LHHHHGSHCTSSGDPAEYNLRSRTVLCGTCGQPADKASASGSGAQVGGPISSGSSASSVTVTRSYRSVGG  SGGGSFGDNLVTRSYLLGNSSPRTQSPQNCSIM |
| **Human**  *Homo sapiens* | <https://www.ncbi.nlm.nih.gov/protein/NP_001393920.1> | 664 aa | >NP_001393920.1 lamin isoform A [Homo sapiens]  METPSQRRATRSGAQASSTPLSPTRITRLQEKEDLQELNDRLAVYIDRVRSLETENAGLRLRITESEEVV  SREVSGIKAAYEAELGDARKTLDSVAKERARLQLELSKVREEFKELKARNTKKEGDLIAAQARLKDLEAL  LNSKEAALSTALSEKRTLEGELHDLRGQVAKLEAALGEAKKQLQDEMLRRVDAENRLQTMKEELDFQKNI  YSEELRETKRRHETRLVEIDNGKQREFESRLADALQELRAQHEDQVEQYKKELEKTYSAKLDNARQSAER  NSNLVGAAHEELQQSRIRIDSLSAQLSQLQKQLAAKEAKLRDLEDSLARERDTSRRLLAEKEREMAEMRA  RMQQQLDEYQELLDIKLALDMEIHAYRKLLEGEEERLRLSPSPTSQRSRGRASSHSSQTQGGGSVTKKRK  LESTESRSSFSQHARTSGRVAVEEVDEEGKFVRLRNKSNEDQSMGNWQIKRQNGDDPLLTYRFPPKFTLK  AGQVVTIWAAGAGATHSPPTDLVWKAQNTWGCGNSLRTALINSTGEEVAMRKLVRSVTVVEDDEDEDGDD  LLHHHHGSHCSSSGDPAEYNLRSRTVLCGTCGQPADKASASGSGAQVGGPISSGSSASSVTVTRSYRSVG  GSGGGSFGDNLVTRSYLLGNSSPRTQSPQNCSIM |
| **Guinea pig**  *Cavia porcellus* | <https://www.ncbi.nlm.nih.gov/protein/XP_063105656.1> | 666 aa | >XP_063105656.1 lamin [Cavia porcellus]  METPSQRRATRSGAQATSTPLSPTRITRLQEKEDLQELNDRLAVYIDRVRSLETENAGLRLRITESEEVV  SREVSGIKAAYEAELGDARKTLDSVAKERARLQLELGKVREEFKELKARNTKKEGDLMAAQARLKDLEAL  LNSKEAALSTALSEKRTLESELHDLRGQVAKLEAALGEAKKQLQDEMLRRVDAENRLQTLKEELDFQKNI  YSEELRETKRRHETRLVEIDNGKQREFESRLADALQELRAQHEDQVEQYKKELEKTYSAKLDNARQSAER  NSNLVGAAHEELQQSRIRIDSLSAQLSQLQKQLAAKEAKLRDLEDSLARERDTSRRLLADKEREMADMRA  RMQQQLDEYQELLDIKLALDMEIHAYRKLLEGEEERLRLSPSPTSQQRSRGRTSSHSSQTHGSGGSITKK  RKLEATESRSSFSQHARTSGRVAVEEVDEEGKFVRLRNKSNEDQSMGNWQIKRQNGDDPLLTYRFPPKFT  LKAGQVVTIWASGAGATHSPPTDLVWKAQNTWGCGNSLRTALINSTGEEVAMRKLVRSVTVVEDDEDEDG  DDLLHHHHGSHCSSSGDPAEYNLRSRTVLCGTCGQPADKASASSSAAQLGGSISSGSSASSVTVTRSYRS  VGGSGGGSFGDNLVTRSYLLGNSRARTQSPQNCSIM |
| **Donkey**  *Equus asinus* | <https://www.ncbi.nlm.nih.gov/protein/XP_014692479.1> | 663 aa | >XP_014692479.1 lamin isoform X1 [Equus asinus]  METPSQRRATRSGAQASSTPLSPTRITRLQEKEDLQELNDRLAVYIDRVRSLETENAGLRLRITESEEVV  SREVSGIKAAYEAELGDARKTLDSVAKERARLQLELSKVREEFKELKARNTKKEGDLMAAQARLKDLEAL  LNSKEAALSTALSEKRTLEGELHDLRGQVAKLEAALGEAKKQLQDEMLRRVDAENRLQTLKEELDFQKNI  YSEELRETKRRHETRLVEIDNGKQREFESRLADALQELRAQHEDQVEQYKKELEKTYSAKLDNARQAAER  NSNLVGAAHEELQQSRIRIDSLSAQLSQLQKQLAAKEAKLRDLEDSLARERDTSRRLLAEKEREMAEMRA  RMQQQLDEYQELLDIKLALDMEIHAYRKLLEGEEERLRLSPSPTSQRSRGRASSHSSQTQGGSSITKKRK  LDSTESRSSFSQHARTSGRVAVEEVDEEGKFVRLRNKSNEDQSMGNWQIKRQNGDDPLLTYRFPPKFTLK  AGQVVTIWAAGAGATHSPPSDLVWKAQNTWGCGNSLRTALINSTGEEVAMRKLVRSVTVVEDDEDEDGDD  LIHHHHGSHCSSSGDPAEYNLRSRTVLCGTCGQPADKASASSSGAQVGGSISSGSSASSVTVTRSYRSVG  GSGGGSFGDNLVTRSYLLGNSSPRTQNQNCSIM |
| **Pig**  *Sus scrofa* | <https://www.ncbi.nlm.nih.gov/protein/NP_001104727.2> | 664 aa | >NP_001104727.2 prelamin-A/C [Sus scrofa]  METPSQRRATRSGAQASSTPLSPTRITRLQEKEDLQELNDRLAVYIDRVRSLETENAGLRLRITESEEVV  SREVSGIKSAYEAELGDARKTLDSVAKERARLQLELSKVREEFKELKARNTKKEGDLMAAQARLKDLEAL  LNSKEAALSTALSEKRTLEGELHDLRGQVAKLEAALGEAKKQLQDEMLRRVDAENRLQTLKEELDFQKNI  YSEELRETKRRHETRLVEIDNGKQREFESRLADALQELRAQHEDQVEQYKKELEKTYSAKLDNARQSAER  NSNLVGAAHEELQQSRIRIDSLSAQLSQLQKQLAAKEAKLRDLEDSLARERDTSRRLLADKEREMAEMRA  RMQQQLDEYQELLDIKLALDMEIHAYRKLLEGEEERLRLSPSPTSQRSRGRASSHSSQTQSGGSVTKKRK  LESSESRSSFSQHARTSGRVAVEEVDEEGKFVRLRNKSNEDQSMGNWQIKRQNGDDPLLTYRFPPKFTLK  AGQVVTIWAAGAGATHSPPADLVWKSQNTWGCGNSLRTALINSTGEEVAMRKLVRSVTMIEDDEDEDGDD  LLHHHHGSHGSSSGDPAEYNLRSRTVLCGTCGQPADKASASSSGAQVGGSISSGSSASSVTVTRSYRSVG  GSGGGSFGDNLVTRSYLLGNSRPRTQSPQNCSIM |
| **Dog**  *Canis lupus familiaris* | <https://www.ncbi.nlm.nih.gov/protein/NP_001274080.1> | 665 aa | >NP_001274080.1 lamin [Canis lupus familiaris]  METPSQRRATRSGAQASSTPLSPTRITRLQEKEDLQELNDRLAVYIDRVRSLETENAGLRLRITESEEVV  SREVSGIKAAYEAELGDARKTLDSVAKERARLQLELSKVREEFKELKARNTKKEGDLMAAQARLKDLEAL  LNSKEAALSTALSEKRTLEGELHDLRGQVTKLEAALGEAKKQLQDEMLRRVDAENRLQTLKEELDFQKNI  YSEELRETKRRHETRLVEIDNGKQREFESRLADALQELRAQHEDQVEQYKKELEKTYSAKLDNARQSAER  NSNLVGAAHEELQQSRIRIDSLSAQLSQLQKQLAAKEAKLRDLEDSLARERDTSRRLLADKEREMAEMRA  RMQQQLDEYQELLDIKLALDMEIHAYRKLLEGEEERLRLSPSPTSQRSRGRASSHSSQTQGTGSITKKRK  LESSESRSSSFSQHARTSGRVAVEEVDEEGKFVRLRNKSSEDQSMGNWQIKRQNGDDPLLTYRFPPKFTL  KAGQVVTIWAAGAGATHSPPTDLVWKAQNTWGCGNSLRTALINSTGEEVAMRKLVRSVTVVEDDEDEDGD  DLLHHHHGSHCSSSGDPAEYNLRSRTVLCGTCGQPADKASASSSGAQVGGSISSGSSASSVTVTRSYRSV  GGSGGGSFGDSLVTRSYLLGSSSPRTQSPQNCSIM |
| **Goat**  *Capra hicus* | <https://www.ncbi.nlm.nih.gov/protein/XP_017901768.1> | 665 aa | >XP_017901768.1 PREDICTED: lamin [Capra hircus]  METPSQRRATRSGAQASSTPLSPTRITRLQEKEDLQELNDRLAVYIDRVRSLETENAGLRLRITESEEVV  SREVSGIKAAYEAELGDARKTLDSVAKERARLQLELSKVREEFKELKARNSKKEGDLMAAQARLKDLEAL  LNSKEAALSTALSEKRTLEGELHDLRGQAAKLEAALGEAKKQLQDEMLRRVDAENRLQTLKEELDFQKNI  YSEELRETKRRHETRLVEIDNGKQREFESRLADALQELRAQHEDQVEQYKKELEKTYSAKLDNARQSAER  NSNLVGAAHEELQQSRIRIDSLSAQLSQLQKQLAAKEAKLRDLEDSLARERDTSRRLLADKEREMAEMRA  RMQQQLDEYQELLDIKLALDMEIHAYRKLLEGEEERLRLSPSPTSQRSRGRASSHSSQTQSGSSVTKKRK  LESTESRSSFSQHARTSGRVAVEEVDEEGKFVRLRNKSNEDQSMGNWQIKRQNGDDPLLTYRFPPKFTLK  AGQVVTIWAAGAGATHSPPTDLVWKAQNTWGCGNSLRTALINSTGEEVAMRKLVRSVTVVEDDDDEDGDD  LLHHHHGSHAGSSSGDPAEYNLRSRTVLCGTCGQPADKAPASGSGAQVGGSISSGSSASSVTVTRSYRSV  GGSGGGSFGDSLVTRSYLLGNSRPRTQSPQNCSIM |

**Table S3 – Lamin B1 and B2**

Table S3: Input NCBI accession numbers and FASTA amino acid sequences for multiple sequence alignment between ten mammal orthologs and the human lamin B1 and lamin B2 protein. “aa” = Amino acid, the length of sequence per ortholog is stated left to the FASTA. Results see Figure S3.

| **SPECIES** |  | **FASTA Lamin B1 ortholog** |  | **FASTA Lamin B2 ortholog** |
| --- | --- | --- | --- | --- |
| **Mongolian gerbil**  *Meriones unguiculatus* | 588 aa | >XP_021515085.2 LOW QUALITY PROTEIN: lamin-B1 [Meriones unguiculatus]  MATATPXVPQRAGSRVSAPSTPLSPTRLSRLQEKEELRELNDRLAVYIDKVRSLETENSALQLQVTEREE  VRGRELTGLKALYETELADARRALDDTARERAKLQIELGKFKAEHDQLLLNYAKKESDLNGAQMKLREYE  AALNSKDAALATALGDKKSLEGDLEDLKDQIAQLEASLSAAKKQLADETLLKVDLENRCQSLTEDLEFRK  NMYEEEINETRRKHETRLVEVDSGRQIEYEYKLAQALHEMREQHDAQVRLYKEELEQTYHAKLENARLSS  EMNTSTVNSAREELMESRMRIESLSSQLSNLQKESRACLERIQELEDMLAKEKDNSRRMLSDKEREMAEI  RDQMQQQLNDYEQLLDVKLALDMEISAYRKLLEGEEERLKLSPSPSSRVTVSRASSSRSVRTTRGKRKRV  DVEESEASSSVSISHSASATGNVCIEEIDVDGKFIRLKNTSEQDQPMGGWEMIRKIGDTSVSYKYTSRYV  LKAGQTVTVWAANAGVTASPPTDLIWKNQNSWGTGEDVKVMLKNSQGEEVAQRSTVFKTTIPEEEEEEEE  EPLGVAVEEERFHQQGASRASNRSCAIM | 612 aa | >XP_021504579.1 lamin-B2 isoform X1 [Meriones unguiculatus]  MSAPHPGSRGDPRAAASAMASVPPRAGPATPLSPARLSRLQEKEELRELNDRLAHYIDRVRALELENDRL  LLRISEKEEVTTREVSGIKALYESELADARRVLDETARERARLQIEMGKLQAELDEARKSAKKREGELTV  AQGRVKDLESLFHRSEAELTAALSDKHSLETDVAELRAQLAKAEDGHAVAKKQLEKETLMRVDLENRCQS  LQEELGFSKSVFEEEVRETRRRHERRLVEVDSSRQQEYDFKMAQALEDLRSQHDEQVRLYRLELEQTYQA  KLDHAKLSSDQNDKAASAAREELKEARMRVESLSYQLSGLQKQASAAEDRIRELEEALAGERDKFRKMLD  AKEQEMTEVRDAMQQQLAEYQELLDIKLALDMEISAYRKLLEGEEERLKLSPSPSSRVTISRATSSSSSS  GVGVSAGRGRGKRRRVETEDTPASHSSSSSMSSGSRLAQQAVATGVVSIDEVDLQGRFVRLKNASDKDQS  LGNWRIKRQVLDGEDISYKFTPKYVLRAGQTVTVWAAGAGVAHSPPSTLVWKSQSSWGSGESFRTVLVNA  DGEEVAVQAVKQSATQGSENGEEEEEEAEFGEEDLFHQQGDPRTTSRGCRLM |
|  |  | LMNB1: <https://www.ncbi.nlm.nih.gov/protein/2599547771>  LMNB2: <https://www.ncbi.nlm.nih.gov/protein/XP_021504579.1> | | |
| **Rabbit**  *Oryctolagus cuniculus / Lepus europaeus^[[1]](#footnote-1)^* | 587 aa | >XP_002710222.1 lamin-B1 [Oryctolagus cuniculus]  MATATPVPPRSGSRAGAPTTPLSPTRMSRLQEKEELRELNDRLAVYIDKVRSLETENSALQLQVTEREEV  RGRELTGLKALYETELADARRALDDTARERAKLQIELGKCKAEHDQLLLNYAKKESDLNGAQIKLREYEA  ALNSKDAALATALGDKKSLEADLEDLKDQIAQLEASLAAAKKQLADETLLKVDLENRCQSLTEDLEFRKN  MYEEEINETRRKHETRLVEVDSGRQIEYEYKLAQALHEMREQHDAQVRLYKEELEQTYHAKLENARLSSE  MNTSSVNSAREELMESRMRIESLSSQLSNLQKESRACLERIQELEDLLAKERDNSRRLLSDKEREMAEIR  DQMQQQLNDYEQLLDVKLALDMEISAYRKLLEGEEERLKLSPSPSSRVTVSRASSSRSVRTMRGKRKRVD  VEESEASSSVSISHSASATGNVCIEEIDVDGKFIRLKNTSEQDQPMGGWEMIRKIGDTSVSYKYTSRYVL  KAGQTVTIWAANAGVTASPPTDLIWKNQNSWGTGEDVKVILKNSQGEEVAQRSTVFKTTLPEEEEEEEEE  AAGLVTEEELFHQQGTPRASNRSCAIM | 624 aa | >XP_062040903.1 lamin-B2 isoform X1 [Lepus europaeus]  MSAPSPGRRRRRRRREPRRPRAAAMATPLPGRAGGPATPLSPTRLSRLQEKEELRELNDRLAHYIDRVRA  LELENDRLLLRISEKEEVTTREVSGIKALYEAELADARRVLDETARERARLQIEAGKLQAELEEASKSAK  KREGELSTAQARLKDLEALLHRSEAELAAALSDKRGLESAAAELRAQLAKAEDGHAVAKQQLEKETLMRV  DLENRCQSLQEELDFRKSVFEEEVRETRRRHERQLVEVDSSRQQEYDFKMAQALEELRGQHDEQVRLYRQ  ELEQTYQAKLDSAKLSSDQKDKAASAAREELQEARMRVESLSYQLSGLQKQASAAEDRIRELEESAAGER  DKFRKMLDAKEQEMAAMRDLVQQQLAEFQELLDVKLALDMEISAYRKLLEGEEERLKLTPSPSSRVTVSR  ASASSSGSSVSTAGRAGRGKRRRLEAEELVGSGPGAGSGSSFRLAQQASATGAVGIEEVDLEGRFVQLKN  SSDKDQPLGNWRIKRQVLEGPEIAYKFTPKYVLRAGQTVTVWAAGAGVAHSPPSTLVWKSQSSWGAGESV  RTVLLNADGEEVAMRTVKQSAAAARESGDGEEDGEEDEEAALGEEDLFHQQGDPRTTSRGCRVM |
|  |  | LMNB1: <https://www.ncbi.nlm.nih.gov/protein/291387267>  LMNB2: <https://www.ncbi.nlm.nih.gov/protein/XP_062040903.1> | | |
| **Rat**  *Rattus norvegicus* | 587 aa | >NP_446357.2 lamin-B1 [Rattus norvegicus]  MATATPVQQRAGSRASAPATPLSPTRLSRLQEKEELRELNDRLAVYIDKVRSLETENSALQLQVTEREEV  RGRELTGLKALYETELADARRALDDTARERAKLQIELGKFKAEHDQLLLNYAKKESDLSGAQIKLREYEA  ALNSKDAALATALGDKKSLEGDLEDLKDQIAQLEASLSAAKKQLADETLLKVDLENRCQSLTEDLEFRKN  MYEEEINETRRKHETRLVEVDSGRQIEYEYKLAQALHEMREQHDAQVRLYKEELEQTYHAKLENARLSSE  MNTSTVNSAREELMESRMRIESLSSQLSNLQKESRACLERIQELEDMLAKERDNSRRMLSDKEREMAEIR  DQMQQQLNDYEQLLDVKLALDMEISAYRKLLEGEEERLKLSPSPSSRVTVSRASSSRSVRTTRGKRKRVD  VEESEASSSVSISHSASATGNVCIEEIDVDGKFIRLKNTSEQDQPMGGWEMIRKIGDTSVSYKYTSRYVL  KAGQTVTVWAANAGVTASPPTDLIWKNQNSWGTGEDVKVVLKNSQGEEVAQRSTVFKTTIPEEEEEEEEE  PIGVAIEEERFHQQGTPRASNKSCAIM | 616 aa | >NP_001292164.1 lamin-B2 [Rattus norvegicus]  MSAPDPGSSADPPAAATAMASLPPRAGSATPLSPTRLSRLQEKEELRELNDRLAHYIDRVRALELENDKL  LLRISEKEEVTTREVSGIKTLYESELADARRVLDETARERARLQIEIGKVQAELEEAKKSAKKREGELTV  ARGRVKDLESLFHRSEAELAAALGDKHVLETEVAELRAQLAKAEDGHAVAKKQLEKETLMRVDLENHCQS  LQEELAFSKSVFEEEVQETRRRHERHLVEVDSSRQQEYDFKIAQALEELRSQHDEQVRLYRLELEQTYQA  KLDNTKLISDQNDKAARAAREELKEARMRVESLSYQLSGLQKQASAAEDHIRELEEALAGERDKFRNMLD  AKEQEMAEVRDAMQQQLAEYQELLDIKLALDMEICSYRKLLEGEEERLKLSPSPSSRVTISLATSSSSSS  SSSGVGMSLGRGRSKRQRLETEDTPGSPSSASSVSSGSRLAQQAGATGVVNIDEVDLEGRFVRLKNSSDK  DQSLGNWRIKKQVLEGEDIAYKFTPKYVLRAGQTVTVWAAGAGATHSPPSTLVWKNQSSWGSGENFRTVL  VNADGEEVAVQAAKPPSVQGHESREEEEEAEAEFGEEDLFHQQGDPRTTSRGCRMM |
|  |  | LMNB1: <https://www.ncbi.nlm.nih.gov/protein/NP_446357.2>  LMNB2: <https://www.ncbi.nlm.nih.gov/protein/NP_001292164.1> | |  |
| **Mouse**  *Mus musculus* | 588 aa | >NP_034851.2 lamin-B1 [Mus musculus]  MATATPVQQQRAGSRASAPATPLSPTRLSRLQEKEELRELNDRLAVYIDKVRSLETENSALQLQVTEREE  VRGRELTGLKALYETELADARRALDDTARERAKLQIELGKFKAEHDQLLLNYAKKESDLSGAQIKLREYE  AALNSKDAALATALGDKKSLEGDLEDLKDQIAQLEASLSAAKKQLADETLLKVDLENRCQSLTEDLEFRK  NMYEEEINETRRKHETRLVEVDSGRQIEYEYKLAQALHEMREQHDAQVRLYKEELEQTYHAKLENARLSS  EMNTSTVNSAREELMESRMRIESLSSQLSNLQKESRACLERIQELEDMLAKERDNSRRMLSDREREMAEI  RDQMQQQLSDYEQLLDVKLALDMEISAYRKLLEGEEERLKLSPSPSSRVTVSRASSSRSVRTTRGKRKRV  DVEESEASSSVSISHSASATGNVCIEEIDVDGKFIRLKNTSEQDQPMGGWEMIRKIGDTSVSYKYTSRYV  LKAGQTVTVWAANAGVTASPPTDLIWKNQNSWGTGEDVKVILKNSQGEEVAQRSTVFKTTIPEEEEEEEE  EPIGVAVEEERFHQQGAPRASNKSCAIM | 615 aa | >NP_034852.3 lamin-B2 isoform 1 [Mus musculus]  MSAPHSGNRGDSPAAATSAMASLPPHAGPATPLSPTRLSRLQEKEELRELNDRLAHYIDRVRALELENDR  LLLRISEKEEVTTREVSGIKTLYESELADARRVLDETARERARLQIEIGKVQAELEEARKSAKKREGELT  VAQGRVKDLESLFHRSEAELATALSDKQGLETEVAELRAQLAKAEDGHAVAKKQLEKETLMRVDLENRCQ  SLQEELAFSKSVFEEEVRETRRRHERRLVEVDSSRQQEYDFKMAQALEDLRSQHDEQVRLYRVELEQTYQ  AKLDNAKLLSDQNDKAAHAAREELKEARMRVESLSYQLLGLQKQASAAENHIHELEEALAGERDKFRKML  DAKEQEMTEVRDAMQQQLAEYQELLDIKLALDMEISAYRKLLEGEEERLKLSPSPSSRITISRATSSSSS  SSGVGMSVGQGRGKRRRLETEDTSGSPSRASRVSSGSRLAQQTVATGVVNIDEVDPEGRFVRLKNSSDKD  QSLGNWRIKRQVLEGEDIAYKFTPKYVLRAGQTVTVWAAGAGATHSPPSTLVWKSQTNWGPGESFRTALV  SADGEEVAVKAAKHSSVQGRENGEEEEEEEAEFGEEDLFHQQGDPRTTSRGCRLM |
|  |  | LMNB1: <https://www.ncbi.nlm.nih.gov/protein/NP_034851.2>  LMNB2: <https://www.ncbi.nlm.nih.gov/protein/NP_034852.3> | | |
| **Rhesus monkey**  *Macaca mulatta* | 586 aa | >XP_005557714.3 lamin-B1 [Macaca fascicularis]  MATATPVPPRTGSRAGGPTTPLSPTRLSRLQEKEELRELNDRLAVYIDKVRSLETENSALQLQVTEREEV  RGRELTGLKALYETELADARRALDDTARERAKLQIELGKCKAEHDQLLLNYAKKESDLNGAQIKLREYEA  ALNSKDAALATALGDKKSLEGDLEDLKDQIAQLEASLAAAKKQLADETLLKVDLENRCQSLTEDLEFRKN  MYEEEINETRRKHETRLVEVDSGRQIEYEYKLAQALHEMREQHDAQVRLYKEELEQTYHAKLENARLSSE  MNTSTVNSAREELMESRMRIESLSSQLSNLQKESRACLERIQELEDMLAKEKDNSRRMLTDKEREMAEIR  DQMQQQLNDYEQLLDVKLALDMEISAYRKLLEGEEERLKLSPSPSSRVTVSRASSSRSVRTTRGKRKRVD  VEESEASSSVSISHSASATGNVCIEEIDVDGKFIRLKNTSEQDQPMGGWEMIRKIGDTSVSYKYTSRYVL  KAGQTVTIWAANAGVTASPPTDLIWKNQNSWGTGEDVKVILKNSQGEEVAQRSTVFKTTIPEEEEEEEEA  AGVVVEEELFHQQGTPRTSNRSCAIM | 628 aa | >XP_045234406.2 lamin-B2 isoform X1 [Macaca fascicularis]  MSPPSPGRRREQRRPRAAATMATPLPGRAGGPATPLSPTRLSRLQEKEELRELNDRLAHYIDRVRALELE  NDRLLLKISEKEEVTTREVSGIKALYESELADARRVLDETARERARLQIEIGKLRAELDEVNKSAKKREG  ELTVAQGRVKDLESLFHRSEVELAAALSDKRGLESDVAELRAQLAKAEDGHAVAKKQLEKETLMRVDLEN  RCQSLQEELDFRKSVFEEEVRETRRRHERRLVEVDSSRQQEYDFKMAQALEELRSQHDEQVRLYKLELEQ  TYQAKLDSAKLSSDQNDKAASAAREELKEARMRLESLSYQLSGLQKQASAAEDRIRELEEAMAGERDKFR  KMLDAKEQEMTEMRDVMQQQLAEYQELLDVKLALDMEINAYRKLLEGEEERLKLSPSPSSRVTVSRATSS  SSGGSTSATGRLGRSKRKRLEVEEPLGSGPSVLGTGTGGGGGFHLAQQASASGSVSIEEIDLEGKFVQLK  NNSDKDQSLGNWRIKRQILEGEEIAYKFTPKYVLRAGQMVTVWAAGAGVAHSPPSTLVWKGQSSWGTGES  FRTVLVNADGEEVAMRTVKQSSVVRENENGEEEEEEAEFGEEDLFHQQVLPLEFIPARGSSSRGLCPQ |
|  |  | LMNB1: <https://www.ncbi.nlm.nih.gov/protein/XP_005557714.3>  LMNB2: <https://www.ncbi.nlm.nih.gov/protein/XP_045234406.2> | | |
| **Human**  *Homo sapiens* | 586 aa | >NP_005564.1 lamin-B1 isoform 1 [Homo sapiens]  MATATPVPPRMGSRAGGPTTPLSPTRLSRLQEKEELRELNDRLAVYIDKVRSLETENSALQLQVTEREEV  RGRELTGLKALYETELADARRALDDTARERAKLQIELGKCKAEHDQLLLNYAKKESDLNGAQIKLREYEA  ALNSKDAALATALGDKKSLEGDLEDLKDQIAQLEASLAAAKKQLADETLLKVDLENRCQSLTEDLEFRKS  MYEEEINETRRKHETRLVEVDSGRQIEYEYKLAQALHEMREQHDAQVRLYKEELEQTYHAKLENARLSSE  MNTSTVNSAREELMESRMRIESLSSQLSNLQKESRACLERIQELEDLLAKEKDNSRRMLTDKEREMAEIR  DQMQQQLNDYEQLLDVKLALDMEISAYRKLLEGEEERLKLSPSPSSRVTVSRASSSRSVRTTRGKRKRVD  VEESEASSSVSISHSASATGNVCIEEIDVDGKFIRLKNTSEQDQPMGGWEMIRKIGDTSVSYKYTSRYVL  KAGQTVTIWAANAGVTASPPTDLIWKNQNSWGTGEDVKVILKNSQGEEVAQRSTVFKTTIPEEEEEEEEA  AGVVVEEELFHQQGTPRASNRSCAIM | 620 aa | >NP_116126.3 lamin-B2 [Homo sapiens]  MSPPSPGRRREQRRPRAAATMATPLPGRAGGPATPLSPTRLSRLQEKEELRELNDRLAHYIDRVRALELE  NDRLLLKISEKEEVTTREVSGIKALYESELADARRVLDETARERARLQIEIGKLRAELDEVNKSAKKREG  ELTVAQGRVKDLESLFHRSEVELAAALSDKRGLESDVAELRAQLAKAEDGHAVAKKQLEKETLMRVDLEN  RCQSLQEELDFRKSVFEEEVRETRRRHERRLVEVDSSRQQEYDFKMAQALEELRSQHDEQVRLYKLELEQ  TYQAKLDSAKLSSDQNDKAASAAREELKEARMRLESLSYQLSGLQKQASAAEDRIRELEEAMAGERDKFR  KMLDAKEQEMTEMRDVMQQQLAEYQELLDVKLALDMEINAYRKLLEGEEERLKLSPSPSSRVTVSRATSS  SSGSLSATGRLGRSKRKRLEVEEPLGSGPSVLGTGTGGSGGFHLAQQASASGSVSIEEIDLEGKFVQLKN  NSDKDQSLGNWRIKRQVLEGEEIAYKFTPKYILRAGQMVTVWAAGAGVAHSPPSTLVWKGQSSWGTGESF  RTVLVNADGEEVAMRTVKKSSVMRENENGEEEEEEAEFGEEDLFHQQGDPRTTSRGCYVM |
|  |  | LMNB1: <https://www.ncbi.nlm.nih.gov/protein/NP_005564.1>  LMNB2: <https://www.ncbi.nlm.nih.gov/protein/NP_116126.3> | | |
| **Guinea pig**  *Cavia porcellus* | 585 aa | >XP_063088497.1 lamin-B1 [Cavia porcellus]  MATATPVPPRSGGRAGPTTPLSPTRLTRLQEKEELRELNDRLAVYIDKVRSLETENSALQLQVTEREEVR  GRELTGLKALYETELADARRALDDTARERAKLQIELGKCKAEHDQLLLNYAKKESDLNGAQIKLREYEAA  LNSKDAALATALGDKKSLEGDLEDLKGQIAQLEASLAAAKKQLADETLLKVDLENRCQSLTEDLEFRKSM  YEEEINETRRKHETRLVEVDSGRQIEYEYKLAQALHEMREQHDAQVRLYKEELEQTYHAKLENARLSSEM  NTSTVNSAREELMESRMRIESLSSQLSNLQKESRACLERIQELEDLLAKERDNSRRMLTDKEREMAEIRD  QMQQQLSDYEQLLDVKLALDMEISAYRKLLEGEEERLKLSPSPSSRVTVSRASSSRSVRTTRGKRKRVDV  EESEASSSVSISHSASATGNVCIEEIDVDGKFIRLKNTSEQDQPMGGWEMIRKIGDTAVSYKYTSRYVLK  AGQTVTIWAANAGVTASPPTDLIWKNQNSWGTGEDVKVILRNSQGEEVAQRSTVFKTTIPEEEEEEEEPS  GVDVEKELFHQQGTPRASNRSCAIM | 630 aa | >XP_003461087.2 lamin-B2 [Cavia porcellus]  MSAPSRGRRRDQSRPRAAAAMASPLPSRAGGPATPLSPTRLSRLQEKEELRELNDRLAHYIDRVRALELE  NDRLLLKISEREEVTTREVSGIKALYESELADARRVLDETACERARLQIEMGKLRAELEEVTKSAKKREG  ELTVAQGRVKDLESLFHRSEGELAAALSDKRSLEGEVAELRAQVAKAEDGHAVAKQQLEKETLMRVDLEN  RCQSLQEELDFQKSVCEEEVRESRRRHERRLVEVDSSRQQEYDFKMAQALEELRSQHDQQVQLYRQELEQ  TYQAKLENARLSSDQNDKAASAAREELQEARVRMESLGFQLSSLQSKASAAEERTRELEAALAGEQDKFR  ALLATKEQEMTQMRDAMQQQLAEYQELLDVKLALDVEISAYRKLLEGEEERLKLTPTPSSRVTISRATSS  SSTSSVSTAGRPGRGKRRRVEVEETPGSGSSSSGIGLGSSSSSGGSGSGSGSFHLAQQASATGGVSIEEV  DLEGRFVRLKNHSDKDQSLGNWKIKRQVFEGDEIAYKFTPKYVLRASQTVTVWASGAGVAHSPPSTLVWK  TQSSWGTGKSFRTVLVNADGEEVAVRAVKQTSAVQNGEEDEEEEDAEFGEEDLFHQQGDPRTPARGCRLM |
|  |  | LMNB1: <https://www.ncbi.nlm.nih.gov/protein/XP_063088497.1>  LMNB2: <https://www.ncbi.nlm.nih.gov/protein/XP_003461087.2> | | |
| **Donkey**  *Equus asinus* | 586 aa | >XP_044636453.1 lamin-B1 isoform X1 [Equus asinus]  MATATPVPPRTGSRAGGPATPLSPTRLSRLQEKEELRELNDRLAVYIDKVRSLETENSALQLQVTEREEV  RGRELTGLKALYETELADARRALDDTARERAKLQIELGKFKAEHDQLLLNYAKKESDLNGAQVKLREYEA  ALNSKDAALATALGDKKSLEGELEDLKDQIAQLEASLAAAKKQLADETLLKVDLENRCQSLTEDLEFRKS  MYEEEINETRRKHETRLVEVDSGRQIEYEYKLAQALHEMREQHDAQVKLYKEELEQTYHAKLENARLSSE  MNTSTVNSAREELMESRMRIESLSSQLSNLQKESRACLERIQELEELLAKERDNSRRMLSDKEREMAEIR  DQMQQQLNDYEQLLDVKLALDMEISAYRKLLEGEEERLKLSPSPSSRVTVSRASSSRSVRTTRGKRKRVD  VEESEASSSVSISHSASATGNVCIEEIDVDGKFIRLKNTSEQDQPMGGWEMIRKIGDTSVSYKYTSRYVL  KAGQTVTIWAANAGVTASPPTDLIWKNQNSWGTGEDVKVILKNSQGEEVAQRSTVFKTTIPEEEEEEEAA  PEVVVEEELFHQQGAPGASNRSCAIM | 602 aa | >XP_044608119.1 lamin-B2 isoform X1 [Equus asinus]  MATPLPGRAGGPPTPLSPTRLSRLQEKEELRELNDRLAHYIDRVRALELENDRLLLKISEKEEVTTREVS  GIKTLYESELADARRVLDETARERAQLQIEIGKLHTELEETSKSSKKREGELTVAQGRVKDLESLFHRSE  AELAAALSDKRSLESNVAELRAQLAKAEDGHAVAKKQLEKETLMRVDLENRCQSLQEELDFRKSVFEEEV  RETRRRHERRLVEVDSSRQQEYDFKMAQALEELRAQHDEQVRLYKLELEQTYQAKLDNAKLISDQNDKAA  SAAREELKEALMRVESLSYQLSGLQKQASAAEDRIRELEETMAGERDKFRKLLDAKEREMTEMRDVMQQQ  LAEYQELLDVKLALDMEINAYRKLLEGEEERLKLSPSPSSRITISRAASSSSGSSVSTAVRSGRSKRKRL  EAEESLGSASSAIGSGSSSVSSFHLAQQASASGSVSIEEIDLEGKFVRLKNSSDKDQSLGNWRIKRQILE  GEEIAYKFTPKYVLRAGQTVTVWAAGAGVAHSPPSTLVWKSQNSWGTGESLRAILVNADGEEVAMRTVKQ  SSGMREAENGEEGAEEGADFGEEDLFHQQGDPRTTSRGCRVM |
|  |  | LMNB1: <https://www.ncbi.nlm.nih.gov/protein/XP_044636453.1>  LMNB2: <https://www.ncbi.nlm.nih.gov/protein/XP_044608119.1> | | |
| **Pig**  *Sus scrofa* | 586 aa | >XP_003123930.2 lamin-B1 [Sus scrofa]  MATATPVQSRTGSRAGGPTTPLSPTRLSRLQEKEELRELNDRLAVYIDKVRSLETENSALQLQVTEREEV  RGRELTGLKALYETELADARRALDDTARERAKLQIELGKFKAEHDQLLLNYAKKESDLNGAQIKLREYEA  ALNSKDAALATALGDKKSLESDLEDLKDQIAQLEASLAAAKKQLADETLLKVDLENRCQSLTEDLEFRKN  MYEEEINETRRKHETRLVEVDSGRQIEYEYKLAQALHEMREQHDAQVKLYKEELEQTYHAKLENARLSSE  MNTSTVNSAREELMESRMRIESLSSQLSNLQKESRACLERIQELEDLLAKERDNSRRMLSDKEREMAEIR  DQMQQQLNDYEQLLDVKLALDMEISAYRKLLEGEEERLKLSPSPSSRVTVSRASSSRSVRTTRGKRKRVD  VEESEASSSVSISHSASATGNVCIEEIDVDGKFIRLKNTSEQDQPMGGWEMIRKIGDTSVSYKYTSRYVL  KAGQTVTIWAANAGVTASPPTDLIWKNQNSWGTGEDVKVILKNSQGEEVAQRSTVFKTTIPEEEEEEEEA  AEVAVEEELFHQQGAPRGSNRSCAIM | 630 aa | >XP_003354039.2 lamin-B2 [Sus scrofa]  MSPPSRGRRSEPRGPRAAAAAAAAAAMATPPPGRAGGPATPLSPTRLSRLQEKEELRELNDRLAHYIDRV  RALELENDRLLLKISEKEEVTTREVSGIKTLYESELADARRVLDETARDRARLQIEIGKLGADLEEANKS  AKKREGELMGAQDRIRDLESVFHRSEMELAAALSDKRALENDVAELRAQLAKAEDGHAVAKKQLEKETLM  RVDLENRCQSLQEELAFRKDVFEEEVRETRRRHERRLVEVDSSRQQEYDFKMAQALEELRAQHDEQVRLY  KLELEQTYQAKLDNAKLSSDQNDKAASAAREELKEARMRLESLSYQLSSLQKQASAAEDRIRELEETMAG  ERDKFRKMLDAKEREMTEMRDVMQQQLAEYQELLDVKLALDMEINAYRKLLEGEEERLKLSPSPSSRITI  SRATSSSSGSSVSMAGRSGRSKRKRLEVEEPLGTGSSGIGSGSSTSSSSSFHMAQQASASGSISIEEIDL  EGKFVQLKNSSDKDQSLGNWRIKRQVLEGEEIAYKFTPKYVLRAGQTVTVWAAGAGVAHSPPSTLVWKSQ  NSWGTGGSFRTVLVNADGEEVAIRTVKQSSVAREAENGEEAEDEAAEFGEEDLFHQQGDPRTTSRGCRVM |
|  |  | LMNB1: <https://www.ncbi.nlm.nih.gov/protein/335283403>  LMNB2: <https://www.ncbi.nlm.nih.gov/protein/XP_003354039.2> | | |
| **Dog**  *Canis lupus familiaris* | 586 aa | >XP_531892.4 lamin-B1 isoform X1 [Canis lupus familiaris]  MATATPVPPRSGSRASAPATPLSPTRLSRLQEKEELRELNDRLAVYIDKVRSLETENSALQLQVTEREEV  RGRELTGLKALYETELADARRALDDTARERAKLQIELGKFKAEHDQLVLNYAKKESDLNGAQMKLREYEA  ALNSKDAALATALGDKKSLEGDLEDLKDQIAQLEASLAAAKKQLADETLLKVDLENRCQSLTEDLEFRKN  MYEEEINETRRKHETRLVEVDSGRQIEYEYKLAQALHEMREQHDAQVKLYKEELEQTYHAKLENARLSSE  MNTSTVNSAREELMESRMRIESLSSQLSNLQKESRACLERIQELEDLLAKERDNSRRMLSDKEREMAEIR  DQMQQQLNDYEQLLDVKLALDMEISAYRKLLEGEEERLKLSPSPSSRVTVSRASSSRSVRTTRGKRKRVD  VEESEASSSVSISHSASATGNVCIEEIDVDGKFIRLKNTSEQDQPMGGWEMIRKIGDTSVSYKYTSRYVL  KAGQTVTIWAANAGVTASPPTDLIWKNQNSWGTGEDVKVILKNSQGEEVAQRSTVFKTTIPEEEEEEEEA  PEVVVEEELFHQQGTPRASNRSCAIM | 604 aa | >XP_038285143.1 lamin-B2 isoform X1 [Canis lupus familiaris]  MATPLSGRAGGPATPLSPTRLSRLQEKEELRELNDRLAHYIDRVRALELENDRLLLKISEREEVTTREVS  GIKTLYESELADARRVLDETARERARLQIELGKLRAELEEANKSSKKREGELTVAQGRVKDLESLFHRSE  AELAAALSDKRVLESDLAELRAQLAKAEDGHAVAKKQLEKETLMRVDLENRCQSLQEELDFRKNVFEEEV  RETRRRHERRLVEVDSSRQQEYDFRMAQALEELRSQHDEQVRLYKLELEQTYQAKLDSAKLSSDQNDKAA  SAAREELKEARMRLESLSYQLSGLQKQASAAEDRIRELEETMAGERDKFRKMLDAKEQEMTEMRDVMQQQ  LAEYQELLDVKLALDMEISAYRKLLEGEEERLKLTPSPSSRITVSRATSSSSSSSVSTAGRSGRSKRKRL  EAEESPGPGSSGIGSGSSSSSTTSFHLAQQASASGGVSIEEIDLEGRFVQLKNSSDKDQSLGNWRIKRQV  LEGEEIAYKFTPKYVLRAGQTVTVWAAGAGVAHSPPSTLVWKTQNSWGTGESFRTVLVNADGEEVAMRTV  KQSSVVRETENGEEGEEETAEFGEEDLFHQQGDPRTTSRGCRVM |
|  |  | LMNB1: <https://www.ncbi.nlm.nih.gov/protein/XP_531892.4>  LMNB2: <https://www.ncbi.nlm.nih.gov/protein/XP_038285143.1> | | |
| **Goat**  *Capra hicus* | 586 aa | >XP_005682747.2 PREDICTED: lamin-B1 [Capra hircus]  MATATPVPPRSGSRAGGPATPLSPTRLSRLQEKEELRELNDRLAVYIDKVRSLETENSALQLQVTEREEV  RGRELTGLKALYETELADARRALDDTARERAKLQIELGKFKAEHDQLLLNYAKKESDLNGAQIKLREYEA  ALNSKDAALATALGDKKSLESDLEDLKDQIAQLEASLAAAKKQLADETLLKVDLENRCQSLTEDLEFRKN  MYEEEINETRRKHETRLVEVDSGRQIEYEYKLAQALHEMREQHDAQVKLYKEELEQTYHAKLENARLSSE  MNTSTVNSAREELMESRMRIESLSSQLSNLQKESRACLERIQELEDLLAKERDNSRRMLSDKEREMAEIR  DQMQQQLNDYEQLLDVKLALDMEISAYRKLLEGEEERLKLSPSPSSRVTVSRASSSRSVRTTRGKRKRVD  VEESEASSSVSISHSASATGNVCIEEIDVDGKFIRLKNTSEQDQPMGGWEMIRKIGDTSVSYKYTSRYVL  KAGQTVTIWAANAGVTASPPTDLIWKNQNSWGTGEDVKVILKNSQGEEVAQRSTVFKTTIPEEEEEEEEA  AEVAVEEELFHQQGAPRGSNRSCAIM | 627 aa | >XP_017906266.1 PREDICTED: lamin-B2 [Capra hircus]  MSPPSRGRRSEQRGPRTAAAAAAAMATPQPGRAGGPSTPLSPTRLSRLQEKEELRELNDRLAHYIDRVRA  LELENDRLQLKISEREEVTTREVSGIKTLYEAELADARRVLDETARDRACLQIEMGKLRADLEEATKSAK  KREGELTVAQGRVRDLESVFHRSEAELAAALSDKRALENDVAELRAQLAKAEDGHAVAKKQLEKETLMRV  DLENRCQSLQEELAFRKDVFEEEVRETRRRHERRLVEVDSSRQQEYDFKMAQALEELRAQHDEQVRLYRL  ELEQTYQAKLDNAKLSSDQNDKAASAAREELKEARMRVESLSYQLSGLQKQASAAEDRIRELEETVAGER  DKFRKMLDAKEREMMEVRDMMQQQLAEYQELLDVKLALDMEISAYRKLLEGEEERLKLSPSPSRITVSRA  TTSSSGSSVSTAGRPGRSKRKRLEVEEPPGTGSSGLGSSSSTSSGSSFHLAQQASASGSVSIEEIDLEGR  FVQLKNSSDKDQSLGNWRIKKQVLEGEEISYKFTPKYVLRAGQTVTVWAAGAGVAHSPPSTLVWKSQNSW  GTGESFRTTLVNADGEEVAMRTVKQSSVVRETENGEEGEDEAAEFGEEDLFHQQGDPRTTSRGCRVM |
|  |  | LMNB1: <https://www.ncbi.nlm.nih.gov/protein/926696505>  LMNB2: <https://www.ncbi.nlm.nih.gov/protein/1062986090> | | |

**Figure S3 – B-type lamins (MSA, Phylogenetic trees)**

Figure S3: Result on phylogenetic distances between the species determined strictly from lamin B1 (left) or lamin B2 (right) protein multiple sequence alignment (Clustal Omega 2, inputs see Table S3). The phylogenetic ordering mimics the order of radiation sensitivity for the species (compare Table S1).

LMNB 1 LMNB 2


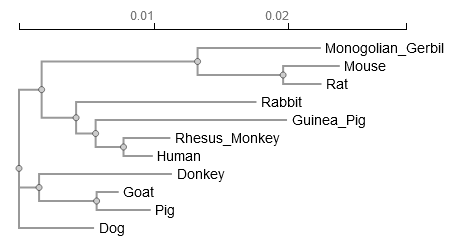

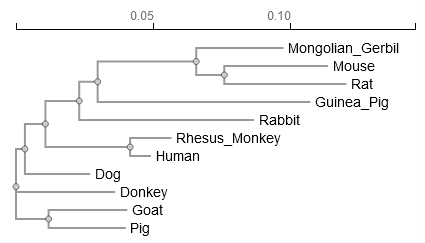


1. *For Oryctolagus cuniculus no lamin B2 ortholog was found, the related Lepus europaeus was taken.* [↑](#footnote-ref-1)
